# Effects of 12-weeks of Brisk Walking on Health-related Physical Fitness, Balance, and Life Satisfaction in Overweight Older Chinese Women: A Cluster Randomized Control Trial

**DOI:** 10.1371/journal.pone.0352243

**Published:** 2026-06-26

**Authors:** Xiaorong Bai, Yuhui Wang, Feifei Chen, Zongqiang Jin, Wensheng Xiao, Soh Kim Geok

**Affiliations:** 1 College of Sports Training, Tianjin University of Sport, Tianjin, China; 2 School of Physical Education, Huzhou Normal University, Huzhou, China; 3 College of Physical Education, Hunan Normal University, Changsha, China; 4 Department of Sports Studies, Faculty of Educational Studies, Universiti Putra Malaysia, Serdang Selangor, Malaysia; Universiti Malaya, MALAYSIA

## Abstract

Obesity among the elderly is primarily the result of a lack of exercise. Many people around the world are concerned about how physical activity can improve the life satisfaction and physical health of overweight seniors. As a result, this study aims to investigate how brisk walking impacts the physical fitness, balance, and overall quality of life of overweight senior women in China. This study design based on cluster randomized control trial. 54 older women between the ages of 60 and 69 were randomly assigned to the brisk walking group (n = 27) or the control group (n = 27). The brisk walking group received three times, (35-60) minutes sessions per week for 12 weeks, while the control group kept their daily routine. Data collection at zero week and twelfth week. Results have presented that brisk walking improved (p < 0.05) cardiorespiratory fitness, flexibility, muscular strength, muscular endurance, balance, and life satisfaction after 12-weeks intervention, except for body composition. Only the chair stand test of muscular endurance showed a significant difference between the brisk walking group and the control group after 12 weeks of training. Nevertheless, the effect size between the groups was increased after 12 weeks of training. In conclusion, brisk walking promotes health-related physical fitness, balance, and life satisfaction among overweight older women after 12 weeks intervention, except body composition.

## Introduction

Population aging constitutes a defining demographic trend in the global population structure [1]. The proportion of older adults in the global population has reached an unprecedented level, with projections of continuous growth in the coming century, and numerous countries are now confronting profound socioeconomic and public health challenges stemming from this demographic shift [2]. With advancing age, age-associated health morbidities grow increasingly prevalent [3], and overweight and obesity among older adults exert a particularly profound adverse impact on physical health: this condition elevates the risk of chronic non-communicable diseases [4], increased fall risk and impaired mobility [5], diminishes subjective life satisfaction [6], and raises overall mortality risk [7]. Robust evidence links higher levels of physical fitness to reduced all-cause mortality, underscoring its role as a modifiable protective factor [8–10]. Life satisfaction, a core dimension of subjective well-being, it serves as a pivotal indicator of overall well-being in older populations [11,12]. However, the majority of current research focuses on life satisfaction and health related physical fitness separately, with comparatively few studies examining the effects of both on the elderly [13,14].

Fostering healthy aging has become a global public health priority, and regular physical activity is a well-recognized cornerstone for maintaining older adults’ physical health and life satisfaction. Despite this consensus, many older adults lack access to low-barrier, evidence-based physical activity interventions [15]. Prior research has demonstrated that brisk walking effectively enhances cardiovascular function and physical endurance—the capacity to sustain daily physical activity and meet unanticipated physical demands [16]. It also strengthens the musculature of the lower extremities, pelvic girdle and lower trunk, preserves the flexibility of major joints, and may ameliorate postural control [17]. Derived from the most fundamental form of ambulation, brisk walking is a moderate intensity aerobic activity that emphasizes standardized posture, controlled speed and sustained duration, with well-documented synergistic benefits for both physical and mental health [18]. Notably, this activity entails minimal physical strain and a low injury risk, rendering it an optimal physical activity option for middle-aged and older adults [19].

Although brisk walking is a low-cost, high-benefit physical activity for older adults [16], the majority of existing studies have focused on fixed moderate-intensity protocols, leaving a critical research gap in the development of intensity-progressive brisk walking interventions tailored to the specific physiological and health needs of vulnerable older adult subgroups [20–22]. Studies have demonstrated that consistent, measurable increases in difficulty force muscles to expand and adapt. Progressive intensity training, on the other hand, can help with conditions like fibromyalgia and sarcopenia by boosting strength and reducing fatigue [23,24]. Two experimental walking intensities have been shown in earlier research: the first involved walking for 15 minutes at a time with 50–70% HR reserve, rising by 5 minutes per week until the patient was able to walk continuously for 30 minutes (week 4) [22]. An alternative strategy is to begin at 40% of the maximum heart rate, progressively raise it to 60% to 80% of the maximum heart rate, and keep it there for the duration of the study [25]. Specifically, there is a dearth of empirical evidence on how a structured, incrementally increasing brisk walking program (with a 5–10% intensity boost every two weeks) impacts the multidimensional health of overweight older Chinese women—a demographic with elevated susceptibility to physical fitness decline, balance impairments and reduced life satisfaction, and one that has been understudied in existing brisk walking intervention research. To address this gap, the present cluster randomized controlled trial investigated the effects of a 12-week progressive intensity brisk walking intervention on three key outcomes (health-related physical fitness, balance and life satisfaction) in this specific population. Findings from this study aim to provide evidence-based, targeted insights for the design of accessible physical activity interventions, and to fill the empirical gap in tailored brisk walking programs for overweight older Chinese women to promote healthy aging.

## Methods

### Study design

A Cluster Randomized Controlled Trial (CRCT) for this study design. Registered an account from ClinicalTrials.gov and got permission (Approval number: NCT04936672, detail has showed in S1 File), and first trial registration in the format 23/06/2021. The CONSORT declaration guided the study’s design and reporting [26]. Sample size calculations typically take into account type error I (α) = 0.05, power (1-*β*) = 0.80 [27], and most studies use G•Power 3.1 to determine the minimum sample size, which comes out to be 52 people [28,29]. A total of 54 individuals should be included in this study, taking into account the effect design and pre-estimated 2–5% dropout rate. Of the 52 (96.3%) were included in the final intention-to-treat (ITT) analysis; two subjects were eliminated because they withdrew from the study in the middle.

The following were the inclusion and exclusion criteria for the two groups: The following conditions must be met for inclusion: older women with sedentary (defined as spending six or more hours a day sitting or lying down with little or no physical activity) [30] or inactive lifestyles (It generally means not meeting the recommended 150 + minutes of weekly moderate-intensity exercise) [31]; the Physical Activity Readiness Questionnaire indicating obvious health [32]；Surgery history and recent physical activity within the previous six months are exclusion factors.

**Fig 1:**
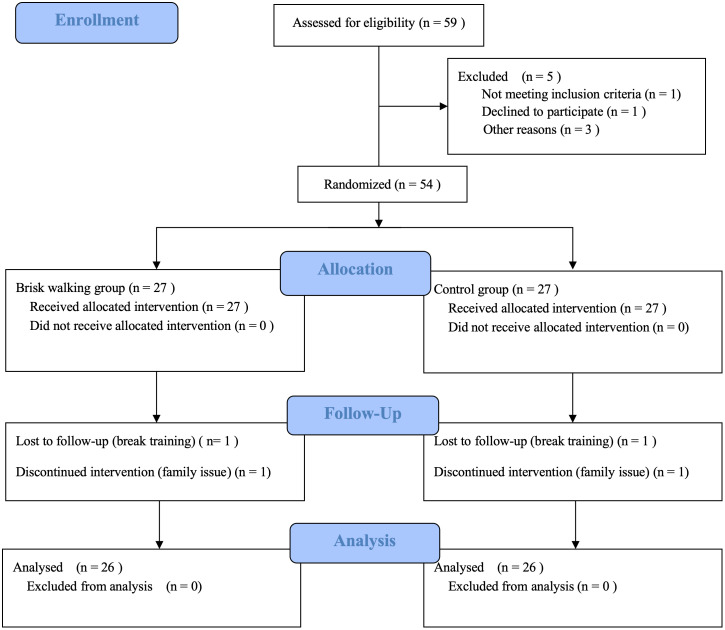
Participants’ flow diagram.

There are no ethical issues associated with this study, all volunteers were fully informed about the procedure and written informed consent was obtained before testing and intervention. Moreover, this study involving human participants were reviewed and approved by Universiti Putra Malaysia Ethics Committee (Protocol number: JKEUPM-2020–296, details were provided in the S3 and S4 Files). Our recruitment period began from June 21st, 2021, to June 24th, 2021. Provide participants with written Informed Consent Forms. The main training started between July 5, 2021, and September 26, 2021. Recruiting from two elderly centers were selected from Puyang city, Henan province, with residents ages ranging from 60 to 69 years, 59 participants were recruited, five participants do not meet the criteria, and finally participants (n = 54) randomly assign into brisk walking (n = 27) and control group (n = 27). The details have shown in Fig 1.

### Blinding and Assignment

This study is CRCT to prevent contamination, the researcher randomly assigned a number for each center and center managers draw lots, with 1 representing the training group and 2 representing the non-training group. Prior to signing the informed consent form, the participants were unaware of the intervention content. The assessor was unaware of the group they were being assessed against.

### Intervention and Control Sections

The intervention followed a standard of exercise by ACSM which is FITT (Frequency, Intensity, Time, Type of exercise) [33]. The Frequency is 3 times per week, intensity is moderate-intensity exercise (50–70% of HRmax) the maximal heart rate (MHR) was calculated with the equation of Tanaka [FCmax = (220-age) *(0.5–0.7)] [34] (Applying the finger touch pulse method throughout training. To maintain the stability of the workout level, the coach regularly reminds us to take our pulse. Using your index and middle fingers, gently press the side of the carotid artery or the inside side of the radial artery for fifteen seconds. To find the heart rate per minute, count the number of pulses and multiply the result by four), and another way of determining moderate intensity is the 1–10 Rating of Perceived Exertion (1–10 RPE), after they have finished cooling down, complete the RPE 10 questionnaire while the monitor watches [35]. Time of the exercise intervention was 12 weeks. Per section time between 35 and 60 minutes, include 10 minutes warm-up and 5 minutes cool-down (Table 1). Type of exercise is brisk walking. The specifics are provided in Table 1. All procedures were carried out at Free Square in the early morning (between 6:00 and 7:00 a.m.), under the supervision of three people who completed the intervention portion. Participants had to refrain from increasing their physical activity, eat healthfully, abstain from alcohol and caffeinated beverages, and obtain plenty of sleep. To make sure the participants were not influenced by other forces, the monitor would ask each participant about their food, sleep patterns, and level of exercise on Fridays of every week. The monitor would just ask about food, sleep, and exercise schedule for the control group. If a member missed more than six sessions or failed to follow the dietary rules three times, they would be eliminated from the program.

**Table 1:** Content of intervention.

Week	Time (min)				Intensity
	Warm-up	Basic content	Cool-down	Duration	HRmax & RPE 10
1st-2nd	10	7.5 min-Rest for 5 min-7.5 min	5	35	HRmax (50%−55%); RPE10 (5-6)
3rd-4th	10	10 min-Rest for 5 min-10 min	5	40	HRmax (55%−60%)；RPE10 (5-6)
5th-6th	10	12.5 min-Rest for 5 min-12.5 min	5	45	HRmax (60%−65%)；RPE 10 (6-7)
7th-8th	10	15 min-Rest for 5 min-15 min	5	50	HRmax (60%−65%)；RPE 10 (6-7)
9th-10th	10	17.5 min-Rest for 5 min-17.5 min	5	55	HRmax (65%−70%)；RPE10 (6-7)
11th-12th	10	20 min-Rest for 5 min-20 min	5	60	HRmax (65%−70%); RPE 10 (6-7)

### Risk Management Strategies Implemented

Typically, three approaches are used to guarantee athletes’ safety: Pre-exercise screening is the first. Before exercising, the Physical Activity Readiness Questionnaire is used to make sure the exercisers are in good health (“No” was the response for every choice). To assure their safety, the physical state of the exercisers is asked about any obvious symptoms, such as nausea or dizziness, prior to the commencement of each exercise. Real-time monitoring comes in second. The coach should choose anti-slip flooring, adequate lighting, and steer clear of high-temperature or high-humidity areas. They should also question the person at any point during the brisk walking procedure if they are feeling fatigued, lightheaded, or experiencing any other pain. Thirdly, the chosen location is 500 meters from a health clinic. The teachers are skilled in cardiac resuscitation, and the fitness centers have basic first aid kits. At the same time, certain criteria—such as severe dyspnea, aberrant heart rate, or chest pain—are established for withdrawal from the trial.

### Outcome measures

The specific operational measures were measured by citing the “Health-related physical fitness assessment manual” [36], “Chinese National Physical Fitness Test Standard Manual” [37], and “Senior Fitness Test Manual Second Edition” [38]. The testing process and standards are carried out in accordance with the standards of ACSM testing [39].

The parameters include cardiorespiratory fitness (systolic pressure, diastolic pressure, resting heart rate, and vital capacity), body compositions (waist-hip-ratio), flexibility (sit and reach and back scratch), muscular fitness (handgrip strength, arm curl, and chair stand), balance (one legged stance with eyes closed), and life satisfaction (satisfaction with life scale [40]).

The baseline (T0) and study end (T12) assessments of these parameters were conducted. Following a brief warm-up, the participants underwent the following steps of the test. Initially, on the day of enrollment, each participant first had to finish all familiarization test. The coach emphasized that no high-intensity exercise should be allowed 24 hours prior to evaluating all the signs, in addition to keeping a light diet, getting enough sleep, and drinking enough water. It is absolutely forbidden to drink alcohol. Thirdly, the tests were carried out over the course of two days, with the experimental group participating on the first day and the control group on the second, all at the same time of day (that is, between 6:00 and 9:00). The research team’s coaches and physical education teachers performed each assessment.

### Data analysis

Descriptive statistics were used to present the demographic characteristics of the total population in the two groups. The homogeneity of two groups for demographics and outcome variables at baseline was examined using two-tailed independent samples t-test. A generalized estimating equation analysis was conducted, followed by Bonferroni correction for pairwise comparison. The effect size (ES) (Cohen d) was interpreted as follows: trivial, < 0.2; small, 0.20 to 0.50; moderate, 0.50 to 0.8; and large, > 0.8 [41]. A *p*-value less than 0.05 was considered statistically significant. IBM SPSS Statistics software for Mac (Version 27; IBM Corporation, Somers, NY, United States) was used for statistical analysis.

## Results

Of the 52 participants who remained in analyses (Fig 1), two of volunteers discontinue attended training caused by them needed to take care of their grandson. the average age was 63.876 ± 2.922 and 63.964 ± 2.441 years, height were 157.827 ± 4.598 and 158.685 ± 4.311, weight were 65.7926 ± 5.792 and 65.801 ± 6.937, education level was 6.885 ± 4.288 and 8.577 ± 4.178, in control group and brisk walking respectively. No significant between-group differences were observed in the participant characteristics at baseline (Table 2).

**Table 2:** Participants’ demographics.

Variables	CG	BW	*p*
	M (SD)	M (SD)	
**Age (year)**	63.876 (2.922)	63.964 (2.441)	0.166
**Height (cm)**	157.827 (4.598)	158.685 (4.311)	0.402
**Weight (kg)**	65.792 (5.314)	65.801 (6.937)	0.463
**Education (years)**	6.885 (4.288)	8.577 (4.178)	0.183

**Note:** CG, control group; BW, brisk walking; M, mean; SD, standard deviation.

**Fig 2:**
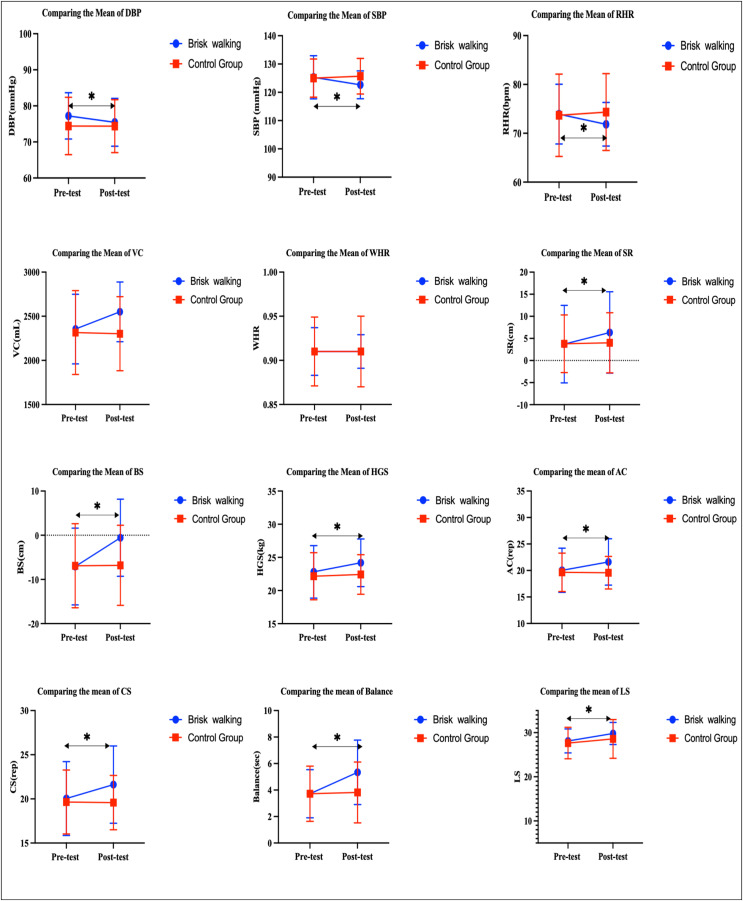
Comparing the mean and standard deviations of brisk walking and control groups before and after 12 weeks of training.

Table 3 summarizes the results of the test batteries. Briefly, after 12 weeks training, brisk walking improved significantly on cardiorespiratory fitness, muscular fitness, flexibility, balance, and life satisfaction, except body composition (Fig 2). Overall, after 12 weeks, brisk walking had a moderate effect on the VC (*p* < 0.05*，*ES = 0.53), CS (*p* < 0.05，ES = 0.68), balance (*p* < 0.05, ES = 0.75), and life satisfaction (*p* < 0.05, ES = 0.65). Moreover, brisk walking had a small training effect on DBP (*p* < 0.05, ES = 0.27), SBP (*p* < 0.05, ES = 0.41), RHR (*p* < 0.05, ES = 0.39), SR (*p* < 0.05, ES = 0. 29), BS (*p* < 0.05, ES = 0.29), HGS (*p* < 0.05, ES = 0.36), AC (*p* < 0.05, ES = 0.37). At 12 weeks, brisk walking group significantly outperformed the control group on measure of CS of muscular fitness (*p* < 0.05). Compared to control group, 12-week brisk walking had a large training effect on the CS (*p* < 0.05, ES = 0.94), moderate training influence on the SBP (*p* > 0.05, ES = 0.54), VC (*p* > 0.05, ES = 0.65), HGS (*p* > 0.05, ES = 0.53), AC (*p* > 0.05, ES = 0.54), and balance (*p* > 0.05, ES = 0.64). Additionally, small training effect on DBP (*p* > 0.05, ES = 0.15), RHR (*p* > 0.05, ES = 0.39), SR (*p* > 0.05, ES = 0.29), BS (*p* > 0.05, ES = 0. 25), and life satisfaction (*p* > 0.05, ES = 0.34).

**Table 3:** Effects of brisk walking on health-related physical fitness, balance, and life satisfaction.

Test battery	Time		Measurement		Between group		Within group d	
							(T0 vs. T12)	
			CG	BW	*p*	*d*	CG	BW
Cardiorespiratory fitness	DBP (mmHg)	T0	74.420 (7.951)	77.230 (6.408)	0.917	0.39	0.01	0.27
		T12	74.380 (7.343)	75.460 (6.629) *	1.000	0.15		
	SBP (mmHg)	T0	125.040 (6.750)	125.310 (7.63)	1.000	0.04	0.10	0.41
		T12	125.690 (6.285)	122.650 (4.931) *	0.288	0.54		
	RHR (bpm)	T0	73.690 (8.423)	73.920 (6.112)	1.000	0.03	0.08	0.39
		T12	74.350 (7.869)	71.850 (4.469) *	0.905	0.39		
	VC (mL)	T0	2315.770 (474.613)	2355.120 (394.849)	1.000	0.09	0.03	0.53
		T12	2302.460 (419.138)	2550.120 (337.729) *	0.917	0.65		
Body composition	WHR	T0	0.910 (0.039)	0.910 (0.027)	1.000	0.01	0.01	0.01
		T12	0.910 (0.040)	0.910 (0.019)	1.000	0.02		
Flexibility	SR (cm)	T0	3.781 (6.530)	3.708 (8.781)	1.000	0.01	0.03	0.29
		T12	4.012 (6.804)	6.346 (9.205) *	1.000	0.29		
	BS (cm)	T0	−6.904 (9.517)	− 7.077 (8.685)	1.000	0.02	0.01	0.29
		T12	−6.800 (9.051)	− 4.558 (8.730) *	1.000	0.25		
Muscular fitness	HGS (kg)	T0	22.158 (3.547)	22.819 (3.955)	1.000	0.18	0.08	0.36
		T12	22.427 (2.984)	24.188 (3.590) *	0.299	0.53		
	AC (rep)	T0	19.650 (3.622)	20.040 (4.181)	1.000	0.01	0.02	0.37
		T12	19.580 (3.075)	21.620 (4.373) *	0.284	0.54		
	CS (rep)	T0	17.580 (3.466)	18.690 (3.564)	1.000	0.32	0.13	0.68
		T12	18.000 (3.072)	21.120 (3.570) *#	0.003	0.94		
Balance	OLS (sec)	T0	3.723 (2.085)	3.720 (1.815)	1.000	0.01	0.04	0.75
		T12	3.818 (2.294)	5.335 (2.432) *	0.110	0.64		
Life satisfaction	LS	T0	27.650 (3.566)	28.120 (2.718)	1.000	0.15	0.23	0.65
		T12	28.580 (4.383)	29.810 (2.498) *	1.000	0.34		

**Note:** CG, control group; BW, brisk walking; T0, pre-intervention test; T12, 12-week post-intervention test; DBP, diastolic blood pressure; SBP, systolic blood pressure; RHR, resting heart rate; VC, vital capacity; WHR, waist-to-hip ratio; SR, sit and reach; BS, back scratch, HGS, handgrip strength; AC, arm curl; CS, chair stand; OLS, one legged stand with eyes closed; LS, life satisfaction; *, within-group differences between T0 and T12, *p* < 0.05; #, BW vs. CG, *p* < 0.05.

## Discussion

This study found that 12 weeks of moderate intensity brisk walking improved health-related physical fitness, balance, and life satisfaction except for body composition. This finding could assist the elderly enhance their physical fitness and quality of life. Furthermore, the overall findings are consistent with prior research.

In this research, 12 weeks of moderate-intensity brisk walking was found to reduce diastolic blood pressure 1.77 mm Hg, systolic blood pressure 2.66 mm Hg, heart rate 2.07 beats/minute, and increase VC 195 ml. All those indicators were improved by brisk walking, this is because when we start exercising, the body’s demand for blood supply and oxygen will increase. During this process, the lungs inhale oxygen, the heart pumps blood, and the blood carries oxygen to all parts of the body through the blood circulation system (Your lungs and exercise). This finding has been confirmed by previous studies, three months of traditional exercise-based lifestyle intervention (Aerobic exercise training, dynamic resistance exercise training, combined aerobic and dynamic resistance exercise training, and isometric exercise training) can result in a reduction in blood pressure of approximately 5 mm Hg systolic [42]. Another study demonstrated a reduction of RHR regardless of any speed walking [43,44]. Distinct walking intensities have distinct effects on RHR. After 12 weeks of brisk walking (60 minutes each session, three times a week, individuals with essential hypertension experienced significant reductions in RHR of 3.6, 8.7 and 11.3 beats per minute, respectively [45]. Previous studies have shown that different walking (fast walking, stepper walking, walking on a treadmill combined with incentive spirometry and Nordic walking) improves VC are consistent with earlier research [46]. Therefore, increasing the intensity and duration of brisk walking every two weeks to improve cardiorespiratory fitness in older overweight women merits greater attention and discussion.

Aging causes in changes to the body’s composition, including a loss of muscle mass, an increase in adiposity, and penetration of muscle fat by fat [47,48]. Obesity is one of the most serious public health issues of the 21st century [49], and is more prevalent among elderly women [50]. The definition of obesity is related to body composition [51]. WHR is a critical metric for assessing obesity. WHR analyzes the risk of diabetes, hypertension, and cardiovascular metabolic disease associated with obesity [52]. Long-term activity (i.e., jogging, walking, Tai Chi, or swimming) can help maintain a healthy body and lower the risk of obesity [53]. According to a study BW have a minor effect on the body fat and body composition of the elderly [54]. This corresponds with the findings of this study. While the benefits of BW in maintaining fitness are apparent, it is still necessary to combine nutrition with targeted activity to minimize WHR. This can help to reduce all-cause mortality caused by rising WHR [55]. Hence, it may take longer persistence and the combination with nutrition to relieve the obesity issue of the elderly.

Aging reduces flexibility due to the natural aging process. This happens for a number of causes, including water loss in tissue and spine, increased joint stiffness, and elasticity loss in the entire tendon and surrounding tissues [56]. Because no stretching occurs before to or during BW, it is doubtful that BW causes changes in flexibility [57]. A study has shown that 12-weeks of brisk walking did not enhance flexibility in the elderly [22]. However, this study found that BW can enhance flexibility (SR and BS) for 12 weeks period, three times a week for 35–60 minutes. Research has demonstrated that stretching exercises aid in the improvement of flexibility [57]. Therefore, the change in flexibility is associated to stretching or not. Stretching links were used before and after the experiment to activate muscle elasticity and change muscle flexibility. Muscle stretching should be incorporated to the walking activity to enhance the flexibility of all regions of the body. As a result, seniors’ flexibility will increase with brisk walking and static muscular stretching before and after exercise.

With increasing age, the strength of the handgrip and quadriceps muscles deteriorates [58]. The handgrip is also associated with multimorbidity, regardless of whether diabetes is included in the morbidity load [59–62]. As the number of comorbid conditions increases, handgrip decreases. The association between handgrip and mortality is frequently utilized to emphasize the critical nature of resistance exercise in physical activity [63]. Thus, one of the most important aspects of keeping older adults physically functional is increasing grip strength through exercise training. The findings of this study suggest that 12 weeks of walking training can improve grip strength by 1.369 kg, similar study has shown that BW (60 minutes, three times a week for 12 weeks at 60% of heart rate reverse) can improve left and right handgrips [64]. Additionally, aging is caused by a variety of functional changes that result in a significant decline in all human capabilities, especially muscular strength and muscular endurance [65]. Muscular strength and endurance are associated with daily mobility. Increases in lean body tissue, bone mineral density, connective tissue strength, anaerobic power, low-back health, and self-esteem are all benefits of physical fitness development [66]. In this regard, our study demonstrates a notable enhancement of 1.58 rep (d = 0.37) in the AC and 2.43 rep (d = 0.68) in the CS indicating its superiority over the practice of before walking exercise. Prior research has shown that 12 weeks of walking training (60 minutes, three times a week for 12 weeks at 60% of heart rate reverse) improve upper and lower muscular strength and endurance explicitly [16]. Thus, BW can be used as an approach to develop muscle strength and endurance in the overweight older women.

Weak balance is a significant factor in the development of falls in the elderly [67]. Falls among the elderly is a significant global public health problem [68]. Fall prevention and balance improvement are global health concerns. Meanwhile, the WHO recommends 150 minutes of moderate-intensity physical exercise each week as beneficial to health and balance [69]. Previous research found that brisk walking promotes balance [70–73]. Similar to the previous study, this study confirmed that brisk walking improves 1.615 seconds (d = 0.75) in the one leg standing of balance. Nonetheless, several investigations found no effect on balance [22,64]. Interventions in studies do not always result in the desired outcome [74]. Additionally, a limited sample size influences the findings [22]. A study has indicated that 12 weeks leg training improved lower limb strength may lead to balance enhancement in neurologically intact older adults [75]. Many studies have shown that walking can enhance lower limb strength [76–79]. Thus, BW can be used as a kind of exercise to enhance balance, however it requires a long amount of time to retain its effectiveness.

Previous research indicated that after six months of brisk walking, life satisfaction increased. In this study just 12-weeks improved life satisfaction. Hence, shorter period promote life satisfaction in older adults is possible. Life satisfaction is associated with health, mortality, and successful aging in older adults, particularly as the years’ pass [80]. Previous research established a positive correlation between physical activity and life satisfaction and happiness in older [81]. Life satisfaction is a measure of well-being that incorporates mood, relationship satisfaction, goal attainment, self-concepts, and self-perceived ability to cope with daily life [82]. Additionally, satisfaction with one’s physical appearance, social or familial relationships, and financial situation may all contribute to overall life satisfaction [81]. When it comes to aging, the elderly’s health situation has the greatest impact on life satisfaction [83]. Thus, it is critical in this field to analyze a variety of criteria to determine the influence of BW on life satisfaction.

### Limitation and Recommendation for Future Study

This study has several limitations that should be considered when interpreting the results.

Firstly, antihypertensive medication use was not included as an inclusion or exclusion criterion, meaning participants may have been on stable antihypertensive regimens throughout the 12-week intervention period. Although all blood pressure measurements were conducted under standardized conditions in line with American Heart Association guidelines, residual confounding from antihypertensive medications may still influence baseline blood pressure levels and post-intervention changes. This confounding factor could affect the interpretation of blood pressure outcomes, as medication use directly impacts blood pressure regulation and may mask or alter the true effects of the progressive intensity brisk walking intervention on blood pressure. Future studies should consider stratifying participants by antihypertensive medication use or controlling for this variable in statistical analyses to reduce potential confounding and enhance the validity of blood pressure-related findings.

Secondly, the exercise intensity in this study was a 5% increase in maximum heart rate every two weeks and a 5-minute increase in duration. However, the study only measured the change in the relevant indicators after 12 weeks and did not measure the effect of the change cycle. Subsequent studies can quantify the impact indicators at various time intervals and track their changes.

Thirdly, there are no comparisons with the exercise group that does not change moderate intensity during training. Only the control and experimental groups are examined in this study. It is therefore difficult to predict which form of intervention will have the biggest effect on the health of older adults. Nevertheless, the results of this study have a significant influence on the health of the elderly, and further investigation might evaluate the effectiveness of various intervention strategies by contrasting the two design approaches.

## Conclusion

In conclusion, a 12-week progressive brisk walking intervention significantly improves health-related physical fitness, balance, and life satisfaction among overweight older women. These improvements are clinically meaningful, as better physical fitness and balance directly reduce fall risk and maintain functional independence, while higher life satisfaction reflects enhanced overall well-being in later life. The structured design of this program — involving moderate-intensity exercise with a 5% intensity increase every two weeks and gradual extension of walking duration — appears both safe and effective for this vulnerable population. Accordingly, progressive brisk walking protocols can be strongly recommended as a feasible, low-barrier exercise strategy for health practitioners, community aging programs, and older adults themselves. Widespread implementation of such evidence-based interventions holds meaningful long-term public health value for supporting healthy aging and managing the challenges of population aging worldwide.

## Supporting information

S1 File Trial Study Protocol. (PDF)

S2 File CONSORT-2010-Checklist. (DOCX)

S3 File Ethic. (PDF)

S4 File Research Protocol for Ethic. (DOCX)
