# Supplementary material for: Effects of 12-weeks of Brisk Walking on Health-related Physical Fitness, Balance, and Life Satisfaction in Overweight Older Chinese Women: A Cluster Randomized Control Trial
Source: PLoS One. 2026 Jun 26;21(6):e0352243. doi: 10.1371/journal.pone.0352243 (PMC13308794; doi:10.1371/journal.pone.0352243)
Supplement: S1 File — (PDF) [file pone.0352243.s001.pdf]

ClinicalTrials.gov PRS DRAFT Receipt (Working Version)
Last Update: 08/07/2025 22:44

ClinicalTrials.gov ID: NCT04936672

Study Identification

Unique Protocol ID: JKEUPM-2020-296

Brief Title: Effects of Brisk Walking Combined With Tai Chi Chuan on Health-Related Physical Fitness and Selected Health Parameters Among Older Chinese Women

NOTE : Brief Title should have no more than 120 characters.

Official Title: Effects of Brisk Walking Combined With Tai Chi Chuan on Health-Related Physical Fitness and Selected Health Parameters Among Older Chinese Women

Secondary IDs:

Study Status

Record Verification: September 2021

ERROR : A record for an active study (Overall Recruitment Status is not Completed, Terminated or Withdrawn) must be reviewed, updated and verified at least once per year.

Overall Status: Active, not recruiting

Study Start: July 3, 2021 [Actual]

Primary Completion: October 23, 2021 [Anticipated]

ERROR : Anticipated Primary Completion Date cannot be in the past.

Study Completion: October 24, 2021 [Anticipated]

ERROR : Study Completion Date must be in the future for a study that is Active, not recruiting.

ERROR : Anticipated Study Completion Date cannot be in the past.

Sponsor/Collaborators

Sponsor: Bai Xiaorong

Responsible Party: Sponsor-Investigator
Investigator: Bai Xiaorong [BXiaorong]
Official Title: National secondary sports social instructor
Affiliation: Universiti Putra Malaysia

Collaborators:

Oversight

U.S. FDA-regulated Drug: No

U.S. FDA-regulated Device: No

U.S. FDA IND/IDE: No

Human Subjects Review: Board Status: Approved
Approval Number: JKEUPM-2020-296
Board Name: Universiti Putra Malaysia
Board Affiliation: Jawatankuasa Etika Universiti/UPM
Phone: 03-97691605/1244
Email: jkeupm@upm.edu.my
Address:

Secretariat of JKEUPM
Unit of Ethics Research (Level 5)
Office of the Deputy Vice Chancellor (Research & Innovation)
Universiti Putra Malaysia

Data Monitoring: No

FDA Regulated Intervention: No

Study Description

Brief Summary: Aging is a problem that exists in many countries in the world. China has a large population base and a large number of elderly people. Paying attention to the health of the elderly is a problem that the Chinese government and society have been paying attention to. In order to improve the physical fitness of the elderly, the government encourages the elderly to do more exercise. However, the elderly do not exercise usually, and the way of exercise is relatively simple, which cannot meet the needs of comprehensive improvement of the elderly's physical fitness. For example, endurance, strength, flexibility, and balance are the most important to the elderly's physical fitness. Tai Chi Chuan is very popular in China. 79.36% of people in China choose brisk walking as the basic exercise. These two exercises have no venue, equipment requirements, and relatively simple technical movements. They are more convenient for the elderly to implement, and according to the literature It is found that Tai Chi Chaun is very effective in improving the balance and flexibility of the elderly, while brisk walking is very effective in improving endurance, and both Tai Chi and brisk walking can improve the strength of the elderly. Therefore, the combination of Taijiquan and brisk walking in this study can meet the four exercise components of elderly people's comprehensive development of physical fitness, endurance, strength, flexibility, and balance, and through the literature, it is found that the effect of combined exercise is better than that of a single exercise. Therefore, this study is divided into 4 groups, Tai Chi Chuan group, brisk walking group, Tai Chi combination brisk walking group, and control group. The aim of this study is to evaluate the effectiveness of combine brisk walking and Tai Chi Chuan (BWTCC) on health-related physical fitness and selected health parameters among older Chinese women. The specific objectives of these studies are as follows:

1. To evaluate the effectiveness of brisk walking (BW), Tai Chi Chuan (TCC), and their combination (BWTCC) on health-related physical fitness (Cardiorespiratory fitness, Body composition, Flexibility, Muscular Strength, and Muscular endurance) among older Chinese women at pre-test, post-test and at three (3) months post-intervention.
2. To evaluate the effectiveness of brisk walking (BW), Tai Chi Chuan (TCC), and their combination (BWTCC) on health parameters (Blood pressure, Resting heart rate, Balance, Height, and weight) among older Chinese women at pre-test, post-test and at three (3) months post-intervention.

3. To evaluate the effectiveness of brisk walking (BW), Tai Chi Chuan (TCC), and their combination BWTCC on QoL among older Chinese women at pre-test and post-test.

The following hypothesis # HO1: There are no significant differences among the three intervention groups (BW, TCC and BWTCC) and with the control group on health-related physical fitness (Cardiorespiratory fitness, Body composition, Flexibility, Muscular Strength, and Muscular endurance) among older Chinese women at pre-test and post-test.

HO2: There are no significant differences among the three intervention groups (BW, TCC and BWTCC) and with the control group on health parameters (Blood pressure, Resting heart rate, Balance) among older Chinese women at pre-test and post-test.

HO3: There are no significant differences among the three intervention groups (BW, TCC and BWTCC) and with the control group on QoL among older Chinese women at pre-test and post-test.

NOTE : Brief Summary should have no more than 2500 characters.

Detailed Description:

1. Quality assurance plan that addresses data validation and registry procedures Each group of sites that submit data to researchers has two personnel responsible for the quality of these data. The site coordinator has learned and fully understood all the agreements, policies, procedures, and definitions in the intervention process before the formal intervention process. All personnel involved in measuring and collecting data must learn systematic knowledge before the formal intervention, so as to ensure that all data are valid and accurate.
2. Data checks to compare data entered into the registry against predefined rules for range or consistency with other data fields in the registry.

   Errors in data entry, transfer, or transformation accuracy: These occur when data are entered into the registry inaccurately. Avoidance or detection of accuracy errors can be achieved through upfront data quality checks (such as ranges and data validation checks), reentering samples of data to assess for accuracy (with the percent of data to be sampled depending on the study purpose), and rigorous attention to data cleaning.

   Errors of intention: Avoidance or detection of intentional error can be challenging. Some approaches include checking for consistency of data between sites, assessing screening log information against other sources , and performing onsite audits (including monitoring of source records) either at random or “for cause.”
3. Source data verification to assess the accuracy, completeness, or representativeness of registry data by comparing the data to external data sources (for example, medical records, paper or electronic case report forms, or interactive voice response systems).

   3.1. External audit of registration procedures The researcher will act as an external audit to ensure the quality level of the registry for a specific purpose, and these audits should be conducted according to pre-established standards. Pre-established criteria may include monitoring participant registration rates or sites that require attention from previous audit results, or monitoring may be based on on-site experience level, serious adverse event reporting rates, or identified issues. This approach can range from reviewing procedures and interviews with on-site personnel to checking and screening logs, to monitoring the process of recording the program.

3.2. Assurance of system integrity and safety All aspects of the data management process should follow a strict life cycle approach for system development and quality management. Each process is clearly defined and documented.

4. Data dictionary that contains detailed descriptions of each variable used by the registry, including the source of the variable, coding information is used (for example, World Health Organization Drug Dictionary, MedDRA), and normal ranges if relevant.

   The variables involved here mainly include independent variables, dependent variables, independent variables include brisk walking, Tai Chi Chuan, and dependent variables include health-related physical fitness, health-related parameters, and quality of life.

   The independent variable has no coding information, only the type of exercise, and the corresponding digital input information for the dependent variable.

   4.1 Health-Related physical fitness Based on the index of ACSM’s exercise for older adults. Health-Related includes Cardiorespiratory Fitness, Body Composition, Flexibility, Muscular Strength, and Muscular Endurance.

   Cardiorespiratory Fitness is usually quantified by the maximum oxygen uptake (VO2max), which is a maximum capacity (exhaustive) test that lasts 5-15 minutes. VO2max, the maximum test has many ways, including bicycle, dynamometer, step, walking/jogging test. Generally speaking, these tests use sub-maximal exercise heart rate, age to predict maximum heart rate (usually 220-age), power estimation VO2max Submaximal tests are best to use the original plan, but it can be based on elderly subjects Exercise capacity or limiting factors are revised to suit the use (Simpson Jr., 2010).

   Body composition measurement method can use simple measurement indicators such as height and weight [body mass index (BMI)], girth, waist-to-hip ratio and/or skinfold thickness, and only requires limited equipment; and Indicators, such as the use of underwater weighing, X-ray technology or ingestion of isotopes, require very high-end measuring equipment: the latter method is usually considered the "gold standard for body composition". For most trainers, limited equipment and easy operation are feasible (Rich, 2004b).

   Flexibility tensility bendiness adaptability adjustability open-endedness openness to change changeability freedom latitude mobility variability fluidity versatility wriggle room wiggle room pliability suppleness pliancy malleability moldability stretchability workability limberness ductility plasticity elasticity stretch stretchiness whippiness springiness spring resilience give bounce bounciness willingness to compromise accommodation adaptability amenability cooperation tolerance forgivingness (Wojtek J. dhbdzko Zajko, 2017).

   The definition of muscular strength is the maximal force that can be generated by a specific muscle or muscle group. Muscle strength is specific to the muscle group. type of contraction (static or dynamic; concentric or eccentric), the speed of the contraction, and the joint angle being tested (Rich, 2004b).

   The definition of muscular endurance is the ability of a muscle group to execute repeated contractions over a period of time sufficient to cause muscular fatigue or to maintain a specific percentage of the maximum voluntary contraction for a prolonged period of time (Rich, 2004b).

4.2 Health parameters Health parameters are health-related impact indicators (Johar, 2012). In this study, resting heart rate, blood pressure, and balance were selected.

Resting heart rate#RHR# National Cancer Institute refers to in medicine, the number of times the heart beats within a certain time period, usually a minute. The heart rate can be felt at the wrist, side of the neck, back of the knees, top of the foot, groin, and other places in the body where an artery is close to the skin. The resting heart rate is normally between 60 and 100 beats a minute in a healthy adult who is at rest. Measuring the heart rate gives important information about a person’s health. Also called a pulse.

Resting heart rate (RHR) is positively related to mortality. Regular exercise causes a reduction in RHR. Exercise—especially endurance training and yoga—decreases RHR. This effect may contribute to a reduction in all-cause mortality due to regular exercise or sports (Reimers et al., 2018).

Blood pressure National Cancer Institute refers to the force of circulating blood on the walls of the arteries. Blood pressure is taken using two measurements: systolic (measured when the heartbeats, when blood pressure is at its highest) and diastolic (measured between heartbeats, when blood pressure is at its lowest). Blood pressure is written with the systolic blood pressure first, followed by the diastolic blood pressure (for example 120/80).

Age and BMI showed as having the strongest direct effect on blood pressure. Age also had a direct effect on blood glucose level and blood cholesterol level. The effect of age on blood glucose and blood cholesterol was also mediated by BMI. Being of an older age had a direct effect on increased blood glucose, blood cholesterol, and blood pressure, while a higher BMI had a direct effect on increased blood pressure. As women get older, maintaining a normal BMI is beneficial to preventing the increase of their blood glucose, blood cholesterol, and blood pressure (Fikriana & Devy, 2018).

Balance Balance is the ability to maintain the body’s line of gravity over its base of support. A correctly functioning balance system allows a person to maintain a proper vision while moving, to determine the direction and speed of movement, to identify the body’s position in its space, and to make automatic postural adjustments to maintain posture and stability in varying circumstances (Boron & Boulpaep, 2016).

4.3 Quality of Life Quality of life (QOL) is defined by the World Health Organization as ‘individuals' perception of their position in life in the context of the culture and value systems in which they live and in relation to their goals, expectations, standards, and concerns (Martin & Amin, 2017).

5. Standard Operating Procedures to address registry operations and analysis activities, such as patient recruitment, data collection, data management, data analysis, reporting for adverse events, and change management.

   5.1 Recruitment 5.1.1 Recruit group Group selection criteria# Exclusion criteria: classes participating in exercise Four classes were randomly selected by the university for the elderly, among which three classes were the brisk walking group, the Taichi Chuan group, the combination of Tai chi chuan and the brisk walking group, and the other one was the control group.

5.1.2 Screening participants The researcher selects the participants who meet the criteria by asking and asking participants to fill out the PAR-Q questionnaire.

The inclusion and exclusion criteria to screen and then check by the doctor. 5.2 Data collection I.Data collection will start after obtaining the ethical approval, and completed the initial 2-week pilot test.

II.Prior to data collection, the potential participants will be approached and screen for illegibility. Then, they will randomly be assigned into 4 groups; 3 experimental groups (Wk, TCC, TCC+ BW) and 1 control group by stratified sampling.

Cross groups contamination will be control by only selecting 2 clinics in one district. In total 4 districts will be randomly selected. Each district will be randomly assigned to either BW, TCC, TCC+ BW, or a control group. Besides, III.the participants in this study will also be advised not to share any information about their exercise content, time, and other information with their friends and peers.

IV.Four qualified instructors and 4 assistance instructors will be appointed to assist the 4 intervention groups. These instructors will be responsible for monitoring the intervention for the group assign to them. Twenty enumerators that hold a Bachelor's Degree in PE will be appointed to monitor and assist the subjects in each group.

V.All participants are required to accept the intervention at the same time, for example, it is necessary to specify the morning or afternoon of each intervention.

VI.Each experimental group selects 2 people to supervise and remind participants of their exercise status.

VII.1 person as a walking instructor with a national second-level sports social instructor qualification certificate, 1 person as a Tai Chi Chuan skill instructor with a third-level or higher qualification certificate for Tai Chi Chuan.

VIII.Participants in each group established a contact group and were reminded before each exercise. After the exercise, they were required to record a detailed exercise log, such as exercise time and exercise content.

IX.The process of instrument testing: Before the start of data collection, all instruments and documents required for testing will be checked to ensure the validity of the instrument during the test.

X.The test environment, test instruments, and test personnel are consistent. Test each test item 3 times, take the middle value.

XI.The data after the test are all checked and processed by two researchers. 5.3 Change Management A detailed manual of the process: If the international and reference standards change during the intervention, a detailed manual containing all registry strategies, processes, and protocols, as well as a complete data dictionary listing all data elements and their definitions, will be updated regularly. This is for researchers Data collection is crucial.

Infrastructure for continuous training: personnel changes are a common problem. Specific procedures and training infrastructure should be provided at all times to cope with any unexpected changes and replacements of registered personnel or providers who regularly enter data into the record office.

The method of notifying the change: through the mobile phone group, so that the participants can report the relevant data during the exercise to the recorder in time.

6. Sample size assessment

   This was based on a four-group design and the assumption of detecting a moderate effect size (partial 2= 0.06) for a given parameter, a moderate correlation of r=0.5 almost I repeated measures, a level of α=0.05, and power (1-ß) of 0.8 (Timmons et al., 2018). This study will be conducted as a cluster-randomized trial (CRCT) with 4 groups and 20 people per group. The ICC value of 0.01 is used in the calculation of the design effect in this study (Killip et al., 2004). As such the design effect was calculated as below:

   DE = 1 + ρ (m-1) DE = 1 + 0.01 (20-1) = 1.19
   ESS = effective sample size= 80 * 1.19 = 96 (24 subjects per each group)
   According to the literature found in the highly relevant literature of this study, the final dropout rate was 5.75% (Guo LX., 2011). Considering this matter, the final sample size will be considered as 26 subjects per group and the total sample size of the study will be n=104.
7. Plan for missing data to address situations where variables are reported as missing, unavailable, non-reported, uninterpretable, or considered missing because of data inconsistency or out-of-range results.
8. Statistical analysis plan The main methods of data collection in this study were statistical analysis and descriptive analysis.

Data will be analyzed using SPSS software (version 27, IBM Company, Chicago, IL, USA). Statistical significance will be reported at the 0.05 level. The data analysis will be preceded by data cleaning and assumption testing. Descriptive statistics will be used for all variables. Descriptive statistics will be reported as means and standard deviations (SD) or medians and interquartile range (IQR) for continuous variables (according to their distribution) and frequency and percentage for categorical variables. Two Way repeated measure ANOVA/ANCOVA or Generalized Estimating Equation (GEE) model for evaluation of the effectiveness of intervention programs on dependent variables will be used. Before the statistical analysis, assumptions underlying each statistical test will be checked and appropriate measures were taken when assumptions are violated.

NOTE : Detailed Description should have no more than 12000 characters.

Conditions

Conditions: Walking

Keywords: brisk walking
Tai Chi Chuan
combine
health- related physical fitness
health parameter

Study Design

Study Type: Interventional

Primary Purpose: Treatment

Study Phase: N/A

Interventional Study Model: Factorial Assignment

There are four groups, 1 control group, and 3 intervention groups. Three intervention groups include the brisk walking group, the Tai Chi Chuan group, and their combination group.

Number of Arms: 4

Masking: None (Open Label)
Try not to allow communication and contact between participants in each group. Researchers do not participate in training. Researchers do not disclose more research information. Data collection and entry should be operated by professional personnel.

Allocation: Randomized

Enrollment: 100 [Actual]

Arms and Interventions

Arms	Assigned Interventions
Experimental: Brisk walking group<br>Type of exercise is brisk walking. The duration of each exercise is from 35 minutes to 60 minutes, with 5 minutes of training time added every two weeks.<br>The frequency of each exercise is 3 times a week. Total exercise time is 12 weeks. The intensity is 50%-70% of the individual's maximum heart rate, gradually increasing the intensity over time.	Behavioral: Brisk walking and Tai Chi Chuan<br>This study includes four groups, the three experimental groups are brisk walking group, Tai chi chuan group, brisk walking combined with Tai chi chuan group, and control group. Among the three experimental groups and exercise time, exercise intensity and exercise frequency are the same over time.<br>Other Names:<br>• Tai Chi Chuan (only#<br>• Brisk walking (only)<br>• Tai Chi Chuan combined with brisk walking
Experimental: Tai Chi Chuan<br>Type of exercise is Tai Chi Chuan. The duration of each exercise is from 35 minutes to 60 minutes, with 5 minutes of training time added every two weeks.<br>The frequency of each exercise is 3 times a week. Total exercise time is 12 weeks. The intensity is 50%-70% of the individual's maximum heart rate.	Behavioral: Brisk walking and Tai Chi Chuan<br>This study includes four groups, the three experimental groups are brisk walking group, Tai chi chuan group, brisk walking combined with Tai chi chuan group, and control group. Among the three experimental groups and exercise time, exercise intensity and exercise frequency are the same over time.<br>Other Names:<br>• Tai Chi Chuan (only#<br>• Brisk walking (only)<br>• Tai Chi Chuan combined with brisk walking
Experimental: Brisk walking combined with Tai Chi Chuan<br>Type of exercise is brisk walking combine with Tai Chi Chuan. The duration of each exercise is from 35 minutes to 60 minutes, with 5 minutes of training time added every two weeks.<br>The frequency of each exercise is 3 times a week. Total exercise time is 12 weeks. The intensity of brisk walking is 50%-70% of the individual's maximum heart rate, gradually increasing the intensity over time.<br>The intensity of Tai Chi Chuan is 50%-70% of the individual's maximum heart rate.	Behavioral: Brisk walking and Tai Chi Chuan<br>This study includes four groups, the three experimental groups are brisk walking group, Tai chi chuan group, brisk walking combined with Tai chi chuan group, and control group. Among the three experimental groups and exercise time, exercise intensity and exercise frequency are the same over time.<br>Other Names:<br>• Tai Chi Chuan (only#<br>• Brisk walking (only)

Arms	Assigned Interventions
	• Tai Chi Chuan combined with brisk walking
No Intervention: Control Group<br>Keeping their daily life.	

Outcome Measures

Primary Outcome Measure:

1. Body composition measurement by Waist circumference
   Measuring process: The measuring ruler should be a cocoa-curved but inelastic tape ruler. The ruler should be placed on the surface of the skin and should not be pressed against the subcutaneous fat tissue. If you use Gulick elastic handle, its handle can be extended to the same mark every time. The same part should be measured twice. If the difference between the two measurement results is more than 5mm, the scene should be measured again.

   [Time Frame: 5 minutes]
2. Flexibility measurement by sit and reach test
   The sit and reach test is a common measure of flexibility, and specifically measures the flexibility of the lower back and hamstring muscles. Seated forward bend test instrument to measure.

   [Time Frame: 5 minutes]
3. Flexibility measurement by Back Scratch Test
   The Back Scratch Test, or simply the Scratch Test, measures how close the hands can be brought together behind the back.

   Use a meter to measure the overlapping part of your hands.

   [Time Frame: 5 minutes]
4. Muscular strength measurement chair stand test
   The chair stand test is similar to a squat test to measure leg strength, in which participants stand up repeatedly from a chair for 30 seconds.

   The chair stand test is similar to a squat test to measure leg strength, in which participants stand up repeatedly from a chair for 30 seconds.

   equipment required: a straight back or folding chair without arm rests (seat 17 inches/44 cm high), stopwatch

   [Time Frame: 5 minutes]
5. Muscular strength measurement by Arm Curl test
   The aim of this test is to do as many arm curls as possible in 30 seconds. equipment required: 4 pound weight (women, AAHPERD), 5 pound weight (women, SFT). A chair without armrests, stopwatch.

   [Time Frame: 5 minutes]
6. Muscle endurance measurement by knee push-up
   1. The push-up fitness test (also called the press-up test) measures upper body strength and endurance. Place the knees on the floor, the hands below the shoulders, and cross your feet.
   2. Keeping your back straight, start bending the elbows until your chest is almost touching the floor.
   3. Pause and push back to the starting position.
   4. Repeat until the set is complete. No equitment. Scoring: Record the number of correctly completed push-ups.

   [Time Frame: 5 minutes]
7. Muscle endurance measurement by Sit-up
   The sit-up (or curl-up) is an abdominal endurance training exercise to strengthen, tighten and tone the abdominal muscles.

   Technique: Squeeze your stomach, push your back flat and raise high enough for your hands to slide along your thighs to touch the tops of your knees. Don't pull with you neck or head and keep your lower back on the floor.

   [Time Frame: 5 minutes]
8. Cardiorespiratory endurance measurement by Six minutes walking test

Six minutes walking test (6MWT) could be considered as a useful and reliable tool for the assessment and the follow# up of cardiorespiratory response.

Equipment required: measuring tape to mark out the track distances, stopwatch, chairs positioned for resting.

Procedure: The walking course is laid out in a 50 yard (45.72m) rectangular area (dimensions 45 x 5 yards), with cones placed at regular intervals to indicate distance walked. The aim of this test is to walk as quickly as possible for six minutes to cover as much ground as possible. Subjects are set their own pace (a preliminary trail is useful to practice pacing), and are able to stop for a rest if they desire.

[Time Frame: 6 minutes]

Secondary Outcome Measure:

9. BMI (body mass index) measurement by Height and weight
   Height and weight can measure human body shape. The formula is BMI = kg/m2 where kg is a person's weight in kilograms and m2 is their height in metres squared. The healthy range is 18.5 to 24.9. The WHO regards a BMI of less than 18.5 as underweight and may indicate malnutrition, an eating disorder, or other health problems, while a BMI equal to or greater than 25 is considered overweight and above 30 is considered obese (World Healt Organization (WHO), 2006).

   [Time Frame: 2 minutes]

10. Balance measurement by Tinetti-test
   The Tinetti-test (Tinetti et al., 1986)was published by Mary Tinetti (Yale University) to assess the gait and balance in older adults(Berg et al., 1992) and to assess perception of balance and stability during activities of daily living and fear of falling.

   The test requires a hard armless chair, a stopwatch and also, a 15 feet even and uniform walkway. It has 2 sections: one assesses balance abilities in a chair and also in standing; the other assesses dynamic balance during gait on a 15 feet even walkway. The patient is to sit in an armless chair and will be asked to rise up and stay standing. The patient will then turn 360° and then sit back down. This is to test the patients' balance.

   [Time Frame: 5 minutes]

11. Resting heart rate (RHR)
   Resting heart rate (RHR) is the number of times your heart beats per minute (bpm) while at complete rest (Kolloch et al., 2008).

   A healthy resting heart rate for adults is 60 to 80 bpm. Adults with a high level of fitness can have a resting heart rate below 60. Some elite endurance athletes have a resting heart rate below 40 (Almeida & Araújo, 2003).

   [Time Frame: 1 minutes]

12. Blood Pressure
   Measuring blood pressure is important for accurately judging values and screening high blood pressure.

   When the blood pressure of 40-70-year-old people is in the range of 115/75-185/115mmHg, the risk of cardiovascular disease doubles for every 20mmHg increase in systolic blood pressure or 10mmHg increase in diastolic blood pressure. According to JNC7, a systolic blood pressure between 120~139mmHg or a diastolic blood pressure between 80~89mmHg belongs to prehypertension.

   [Time Frame: 1 minutes]

Other Pre-specified Outcome Measures:

13. Quality of life measurement by SWLS
   The Satisfaction With Life Scale (SWLS) is a 7-point Likert style response scale to measure Quality of life.

   Scoring A total score is calculated by adding up the scores for each item. The possible range of scores is 5-35, with a score of 20 representing a neutral point on the scale. Scores between 5-9 indicate the respondent is extremely dissatisfied with life, whereas scores between 31-35 indicate the respondent is extremely satisfied. The following chart provides cutoff scores to be used as benchmarks.

   [Time Frame: 5 minutes]

Eligibility

Minimum Age: 60 Years

Maximum Age: 69 Years

Sex: Female

Gender Based: Yes
Chinese women

Accepts Healthy Volunteers: Yes

Criteria: Inclusion Criteria:

- Less than 30 minutes of moderate-intensity physical activity per week.
- Through the screening of the PAR-Q questionnaire (If one of the questions is answered "yes", the potential participants will be excluded) and the basic physical examination of the elderly by doctors, to determine that the elderly can complete exercise independently.

Exclusion Criteria:

- Subjects participated in other sports training at the same time.
- If the subjects have recently (less than one year) undergone surgery on the knee, elbow, shoulder, etc., has a history of rheumatoid disease or neurological disease, and is still receiving treatment, it will be excluded.
- If the subjects answered “yes” to the PAR-Q questionnaire.

Contacts/Locations

Central Contact Person: Xiaorong Bai, PhD candidate
Telephone: + 86 15039300205
Email: baixiaorong188@gmail.com

Central Contact Backup: Wensheng Xiao, PhD candidate
Telephone: +86 15090213005
Email: xiaowensheng33@gmail.com

NOTE : Contact Degrees should have no more than 12 characters.

NOTE : Contact Degrees should have no more than 12 characters.

Study Officials: NOTE : Study Official is required by the WHO and ICMJE.

Locations: **China, Henan**
Puyang Aged Institution
Puyang, Henan, China
Contact: Shixin Bai, Bachelor 18791254170 313691931@qq.com

IPDSharing

Plan to Share IPD: No

References

Citations: **[Study Results]** ACSM. (2008). ACSM's Health-Related Physical Fitness Assessment Manual. In Medicine & Science in Sports & Exercise.

ACSM's Guidelines for Exercise Testing and Prescription 9th Ed. 2014. (2014). The Journal of the Canadian Chiropractic Association.

	ACSM. (2009). ACSM's Guidelines for exercise Testing and Prescripstion. In Lippincott Williams & Wilkins. https://doi.org/10.1249/MSS.0b013e318213fefb
Links:	
Available IPD/Information:	Type: Study Protocol<br>URL: https://doi.org/10.1249/MSS.0b013e318213fefb<br>The protocol setting and data collection process is based on this reference

U.S. National Library of Medicine | U.S. National Institutes of Health | U.S. Department of Health & Human Services
