# Supplementary material for: Effects of 12-weeks of Brisk Walking on Health-related Physical Fitness, Balance, and Life Satisfaction in Overweight Older Chinese Women: A Cluster Randomized Control Trial
Source: PLoS One. 2026 Jun 26;21(6):e0352243. doi: 10.1371/journal.pone.0352243 (PMC13308794; doi:10.1371/journal.pone.0352243)
Supplement: S2 File — (DOCX) [file pone.0352243.s004.docx]

**Research protocol**

### Project summary

### Obesity among the elderly is primarily the result of a lack of exercise. In several nations, individuals have concerns about how to enhance the physical and emotional health of overweight older people through activity. Thus, this study aimed to investigate at the effects of brisk walking on the physical fitness, balance, and life satisfaction of overweight senior women in China. This study design based on cluster randomized control trial. 54 older women between the ages of 60 and 69 were randomly assigned to the brisk walking group (n = 27) or the control group (n = 27). The brisk walking group received three times, (35-60) minutes sessions per week for 12 weeks, while the control group kept their daily routine. Data collection at zero week and twelfth week. Results have presented that brisk walking improved (p < 0.05) cardiorespiratory fitness, flexibility, muscular strength, muscular endurance, balance, and life satisfaction after 12-weeks intervention, except for body composition. After 12 weeks of training, only Chair stand of muscular endurance was significantly between brisk walking group and control group. Nevertheless, the effect size between the groups was increased after 12 weeks of training. In conclusion, brisk walking promotes health-related physical fitness, balance, and life satisfaction among overweight older women after 12 weeks intervention, except body composition.

### General information

### Protocol title: Effects of Brisk Walking Combined With Tai Chi Chuan on Health-Related Physical Fitness and Selected Health Parameters Among Older Chinese Women; Approval number: NCT04936672, and first trial registration in the format 23/06/2021.

### Funding: This study was supported by A Project Supported by General project of Humanities and Social Sciences Research of Ministry of Education of China, the Scientific Research Fund of Zhejiang Provincial Education Department (Grant No. Y202351082), and the Philosophy and Social Sciences Research Project of Anhui Province (Grant No. AHSKY2022D188).

### Wensheng Xiao, PhD, Lecturer, and Tel: +86 13735107256. He to supervise them to do exercise and guide them to complele training log and check them to rational diet, training site in puyang square, Puyang,Henan province, China.

### Xiaorong Bai, PhD, Lecturer, and Tel: +86 13515728656. She to supervise them to keep daily life routine and check them to rational diet.

### Rationale & background information

Physical activity has been shown to improve the overall health and quality of life (QoL) of the elderly by enhancing their physical and mental health (Awick et al., 2015; Choi et al., 2013; Ordu Gokkaya et al., 2012). Furthermore, the World Health Organization (WHO) reported that people who were physically inactive had a 20% to 30% higher risk of death than those who were physically active. Meanwhile, WHO believes that movement is superior to inactivity and that exercise can improve a person's physical and mental health (WHO, 2020). Thus, supporting healthy aging through physical activity, self-sufficiency, and leisure time becomes a critical public health problem for enhancing an individual's health (Im et al., 2019). However, what exercise method for elderly is comfortable to improve their health? Everyone's perspective is different. Walking is a low-cost, low-impact form of exercise (Bai et al., 2022). Not only does it improve general health and improve QoL, but it also plays a critical role in the prevention and treatment of numerous diseases (Stewart et al., 2017). A study has demonstrated that moderate to high-intensity brisk walking is better than low-intensity walking (Swoap et al., 1994). Though walking is the most studied form of exercise, but suitable intensity of brisk walking for elderly to enhance their health is still unclear (Bai et al., 2022). According to prior studies, brisk walking improved cardiovascular capacity and endurance (stamina) for bodily work and movement in everyday life that also provides reserves for meeting exceptional demands (Steven, 2021). Meanwhile, muscles of the legs, limb girdle and lower trunk are strengthened and the flexibility of their cardinal joints preserved and posture and carriage may improve (Morris & Hardman, 1997). Furthermore, brisk walking emerged from the most basic form of walking. It is an aerobic activity that alternates between brisk walking and running, with a focus on posture, speed, and duration to promote both physical and mental wellness. Brisk walking has the benefit of being less prone to injury and posing low risk, making it a perfect option for middle-aged and older people (Fan, 2018).

Health-related physical fitness and balance relationship with good health and ratio of falls for elderly(ACSM, 2005; Gschwind et al., 2013). Early studies have shown that life satisfaction association with mental health (Lombardo et al., 2018; Michalski et al., 2022). Thus, identifying workouts that have a greater influence on health-related physical fitness, balance, and life satisfaction to prevent and defend against the emergence of health problems warrants further research(Bullo et al., 2018). Consequently, this study is to evaluate the effect of 12-weeks brisk walking on health-related physical fitness, balance, and life satisfaction among overweight older Chinese women.

### Study goals and objectives

This study is to evaluate the effect of 12-weeks brisk walking on health-related physical fitness, balance, and life satisfaction among overweight older Chinese women.

### Study design

The research design was based on a Cluster Randomized Controlled Trial (CRCT). Recruiting from two elderly centers were selected from Puyang city, Henan province, with residents ages ranging from 60 to 69 years, 59 participants were recruited, five participants do not meet the criteria, and finally participants (n = 54) randomly assign into brisk walking (n = 27) and control group (n = 27). The details have shown in Figure 1. The inclusion criteria and exclusion criteria in two groups were as follows: Inclusion criteria: the elderly women who inactivity or sedentary lifestyle (When someone spends six or more hours per day sitting or lying down, and they lack significant physical movement in their daily life); being apparently healthy according to the Physical Activity Readiness Questionnaire. Exclusion criteria: History of surgery and physical exercise in the last six months.


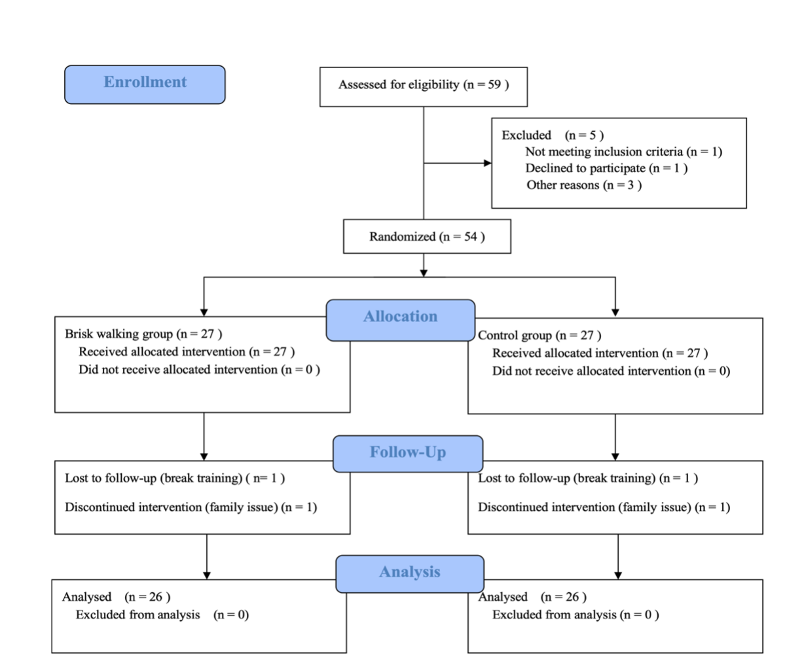


### Methodology

The intervention followed a standard of exercise by ACSM which is FITT (Frequency, Intensity, Time, Type of exercise). The Frequency is 3 times per week, intensity is moderate-intensity exercise (50-70% of HRmax) the maximal heart rate (MHR) was calculated with the equation of Tanaka [FCmax = (220-age) *(0.5-0.7)] , and another way of determining moderate intensity is the 1-10 Rating of Perceived Exertion (1-10 RPE), after they have finished cooling down, complete the RPE 10 questionnaire while the monitor watches. Time of the exercise intervention was 12 weeks. Per section time between 35 and 60 minutes, include 10 minutes warm-up and 5 minutes cool-down. Type of exercise is brisk walking. All procedures were implemented in the morning (Between 6:00 to 7:00 a.m.) at free Square, and three persons supervised them to finish intervention part. Participants were required to avoid engaging in additional physical activity, eat appropriately, avoid caffeinated drinks, alcohol intake, and get excellent sleep. On Fridays of each week, the monitor would inquire about each participant's diet, sleep, and exercise to ensure that the participants were not influenced by external factors. For the control group, the monitor would simply inquire about the situation of diet, sleep, and exercise time. The participant will be removed from the training if they skip more than six sessions or if they don't adhere to the dietary guidelines three times.

**Outcome Measures**

The specific operational measures were measured by citing the “Health-related physical fitness assessment manual” (ACSM, 2010), “Chinese National Physical Fitness Test Standard Manual” (Department of Mass Sports, 2003), and “Senior Fitness Test Manual Second Edition” (Rikli & Jones, 2013). The testing process and standards are carried out in accordance with the standards of ACSM testing (ACSM, 2014). The majority of the testing is done in compliance with the standard; nevertheless, we are only able to guarantee that the test environment and testers for the two test groups are the same because there are no indoor testing facilities available.

The parameters include cardiorespiratory fitness (systolic pressure, diastolic pressure, resting heart rate, and vital capacity), body compositions (waist-hip-ratio), flexibility (sit and reach and back scratch), muscular fitness (handgrip strength, arm curl, and chair stand), balance (one legged stance with eyes closed), and life satisfaction (satisfaction with life scale).

Cardiorespiratory fitness measures include systolic blood pressure (SBP), diastolic blood pressure (DBP), resting heart rate (RHR) and vital capacity (VC). According to the American Heart Association’s standardized protocol (Ali et al., 2018), we measured SBP, DBP, and resting heart rate (RHR) three times for each participant using an electronic sphygmomanometer (Omron HEM-7071A, Japan), after having them sit for at least 5 min. In cases where there was a difference of more than 5 mmHg or 5 beats/min, the two closest values were adopted (Wang et al., 2017). Vital capacity (VC) was measured using a spirometer (Jianmin, GMCS-III types A, Xinheng Oriental Technology Development Co., Ltd, Beijing, China), testing according to the National Physical Health Test standard guidelines of China. The maximum value was recorded after three acceptable maneuver attempts. The middle of the three measurements was used for further data analysis.

Body compositions are assessed by waist-to-hip ratio (WHR). WHR = waist circumference/hip circumference. Waist circumference was measured at a level midway between the lowest rib and the iliac crest using a measuring tape, and hip circumference was measured using the same tape at the widest position of the buttocks, with the tape along a plane parallel to the floor and not compressing the skin, after inhalation and exhalation. Waist and hip circumferences were measured three times for each participant and were accurate to the nearest 0.1 cm, with the average of the three measurements being used for further data analysis.

Flexibility was tested by Sit and Reach (SR) and Back Scratch (BS). Researcher applied sitting body forward bending tester to test SR. The process of testing followed by National Physical Health Test standard guidelines of China. Records are recorded in centimeters, with one decimal place reserved. Take the test twice and get the best score. Back Scratch was tested by a meter rule. The process of testing was followed Chinese National Physical Fitness Test Standard Manual. Practice twice before testing, then test twice and take the best value.

Muscular fitness was tested by Handgrip Strength (HGS), Arm Curl (AC), and Chair Stand (CS). The process of testing was followed Chinese National Physical Fitness Test Standard Manual. Handgrip Strength measuring dominant hand, researcher used a hand-muscle developer (Constant, 14192-709E, Yiwu Chao LAN Import and Export Co., LTD, Yiwu, China). Testing twice and take the best value. The Arm Curl test is conducted on the dominant arm (or stronger side) for 30 seconds while the subject sits in a chair. A 4-pound dumbbell weight was applied in the test. The procedure was adhered to. Second Edition Senior Fitness Test Manual. Chair Stand Test: The participant completed the test in 30 seconds by sitting in the middle of the seat, placing his feet shoulder-width apart. Keep track of how many times the volunteer stood.

One-leg Standing with Eyes Closed: We used the method described in the National Physical Health Test standard guidelines of China. Briefly, upon the assessor’s command, participants were asked to lift the non-dominant leg off the ground and keep their dominant leg vertical; in this position, participants were asked to stand for as long as possible with the time measured to the nearest to 0.01 s using a stopwatch (JinQue, JD-3B, Shanghai Automation Instrument Co., Ltd.). Before the test measurement was conducted, participants practiced 3 trials in the same position as that used in the official measurement. The test was stopped when participants were no longer able to maintain the requirements of the test position.

Questionnaire for Measuring Life Satisfaction (SWLS): SWLS is a 7-point Likert response scale. SWLS scoring is as simple as adding up the scores for each statement. A five-item scale is designed to assess global cognitive judgments about an individual's level of life satisfaction (Riva & Serino, 2020). So, keep in mind that 1 strongly disagrees and 7 strongly agree, and the higher your score, the more satisfied you are with your life. Your overall score indicates that you are very satisfied with 31-35, very satisfied with 26-30, slightly satisfied with 21-25, neutral with 20, slightly dissatisfied with 15-19, dissatisfied with 10-14, very satisfied with 5-9 dissatisfaction.

**Binding and Assignment**

This study is CRCT to prevent contamination, the researcher randomly assigned a number for two centers and center managers draw lots, with 1 representing the training group and 2 representing the non-training group. Prior to signing the informed consent form, the participants were unaware of the intervention. The assessor was unaware of the group they were being assessed against.

### Safety considerations

Walking is actually the safest kind of exercise. Before exercising, we advised participants to put on loose clothing and comfy shoes. Prior to the activity, we issued training manuals and performed training at the same time. Volunteers should keep a training diary after training, and they should notify us right away if there are any problems.

### Follow-up

### Professional sports specialists have monitored our 12-week training regimen to guarantee scientific training techniques and group safety.

### Data management and statistical analysis

The study collected data primarily through statistical analysis and descriptive analysis. All quantitative data were collected during the experiment and analyzed using SPSS software (version 23, IBM Company, Chicago, IL, USA). Statistical significance was determined using two-tailed p-values at the 0.05 alpha level. Data cleaning and hypothesis testing were performed prior to analysis. Researchers use descriptive techniques for quality checking, including identifying missing data, outliers, and coding errors. All variables were analyzed using descriptive statistics. Means and standard deviations (SD) or medians and interquartile range (IQR) were used to describe continuous variables (according to their distribution), and frequency and percentage were used to describe categorical variables. It is helpful to describe each variable individually, including its characteristics, demographic data, and research variables.

Group homogeneity was assessed using one-way ANOVA for continuous variables and Chi-Square test for categorical variables prior to data analysis. Standard empirical statistical test rules for normality are used to ensure that the skewness and kurtosis of continuous variables are between -2 and +2. In the pre-test, the data were checked for homogeneity of variances using Levene's test.

To evaluate the effectiveness of intervention programs on dependent variables, a generalized estimating equation (GEE) model was used. GEE extends the logistic regression model to allow for clustering. This thesis illustrates the analysis of longitudinal data on healthrelated physical fitness, balance, and life satisfaction among the elderly using GEE under various intervention correlations assumptions. Since individuals within the same group tend to be correlated (non-independent), this violates the basic assumptions of individualized randomized controlled trial hypothesis testing and statistical inference. In such cases, analytical methods that can address the effects of cluster design must be used, which can lead to studies that exaggerate intervention effects and produce false positive results. Individual-level analyses preserve the individual observations but still account for the correlation within clusters. Generalized estimating equations (GEE) are commonly used. In addition, the commonly used 57guidelines proposed by Cohen (2013) (small, d=0.2; medium, d=0.5; and large, d=0.8) were used to determine the intervention's effect size.

### Quality assurance

### Control of Extraneous Variables

### The experimental data of this study comes from field tests. Since they are performed in a sports environment (e.g., sports halls, playing fields), the exercise specificity of the test is increased. Therefore, to produce meaningful test results, field-based testing requires considerable thought and careful management to control extraneous irrelevant variables (e.g., time, place, fatigue, main test, program, and training monitoring).

### Ways to Control Variables When Intervening

### Site all the training and test was arranged in Puyang city of Henan province. Training location: Shangyi Square for BW group.Training Time: 6 am-7 am on every Monday, Wednesday, and Friday for each experimental group. Intervention is 12 weeks.Training content: All subjects followed the same procedure for warm-up, main content, and cool down. In the BW group, the technique is based on the book of General Administration of Sports of the People's Republic of China. (2008). Brisk Walking. China Society Press. Training Coach: One coaches and three assistants in experimental groups. One coach and two assistants for each experimental group. One assistant for the control group.Coach Qualification: Coaches are physical education teachers, and they have Second-level Social Instructor Certificate and Physical Education Teacher Certificate in the BW group. Coaches are physical education teachers, and they have Second-level Social Instructor Certificate, Physical Education Teacher Certificate. All the assistants are Physical Education Teachers.Training instruments: Audio and U disk to play training background music. A stopwatch can check the exact time of participants’ exercise. In the BW group, brisk walking-cool walking music was applied and exercise in a circle; For brisk walking, participants exercise in a circle.

### Training control: There are training logs and training attendance to control the participants participate in exercise training. Training log for checking whether participants follow training program or not. Training attendance for coaches to check whether the participants exercise or not for 12 weeks.

### Other control: Participants in both the treatment and control groups were not encouraged to perform any other form of exercise or obtain any help from professionals or read any self-improvement materials during the period of the experimental research to reduce the possibility of maturation among subjects.

### Environmental Control: Environmental factors can have a profound effect on field testing, particularly when performed outdoors due to the weather primarily. Consideration should be given to the effect alterations in the weather may have on the testing environment (e.g., slippery/muddy surfaces) and the sports performer (e.g., temperature extremes). Ideally, tests should be performed in very similar conditions enabling comparisons to be drawn. Therefore, this study should pay attention to environmental conditions when recording the test scores, and the test time can be adjusted in extreme environments.

### Control for Test Protocol

### Before the test, explain the test process and matters needing attention to the subjects, so that the subjects will actively participate in the test with a correct attitude and cooperate with the testers to complete the whole test process. At the same time, it is necessary for the tester to ask the subjects to adhere to the pretest behavior. The test protocol is an important factor that affects the accuracy of the test data. During the test, the test should be carried out in strict accordance with the standardized test protocol. Otherwise, it will not produce meaningful data. Therefore, in the data collection process of this study, the same tester tested the subjects according to the test protocol. Blinding testers, who do not know the names and groups of participants. At the same time, the testers have been trained and tested in strict accordance with the ACSM test guidelines.

### Control for Co-variate

### The model of health-related physical fitness mentioned other factors and genetics that can influence health and health-related fitness. However, recent studies still cannot show the factors clearly and mentioned that genetics are complex and not easy to explain. In order to identify and control co-variate to ensure the validity of the experimental design. A study showed that the Anderson behavior model can bring the influencing factors that affect the physical exercise of the elderly in China. Socio-demographic data include name, birth, height, weight, health concept, income, residence, marital status, educational years, chronic diseases, self-rated health, and health behavior. Thus, it clearly articulates the factors that influence physical activity in older adults and searches relevant literature to discover more co-variate, but that co-variate has already been included in the Socio- demographic data such as age, height, weight, income, gender, health behavior, and education years. Thus, in this study, the co-variate followed the socio-demographic data.

### Expected outcomes of the study

### This study provides strong evidence regarding the benefits of brisk walking for overweight older Chinese women. To maximize the improve of health-related physical fitness, balance, and life satisfaction, all the coaches, elderly and organization can recommend them to exercise by moderate brisk walking to improve their health.

### Dissemination of results and publication policy

### The original contributions presented in the study are included in the article/supplementary material, further inquiries can be directed to the corresponding author/s.

### Duration of the project

### The data collection process is divided into two phases.

### Phase 1: Pre-Intervention

### One month prior to the data collection, the researcher performed thefollowing activities:

### (A) Prior to data collection, all participants signed an informed consent form. Additionally, a two-week pilot study was conducted to view the feasibility of all intervention procedures and the reliability and validity of all testing instruments and questionnaires.

### (B) A two-week meeting was held to brief the three experimental group coaches about the research intervention. Making arrangements for the coaches to meet with participants.

### (C) When meeting with participants. First, explain the ACSM's recommended sports safety procedures to avoid sports-related injuries. Second, coaches to demonstrate how to complete the attendance table. Third, distribution of the sports booklets. Fourth is the introduction of related matters that should be paid attention to during the participation. Finally, inform the participants of the researcher's phone number. If participants have any questions, they can contact the researcher for further explanation and consultation.

### (D) Participants learned the BW process through a video presentation and a manual booklet that includes a step-by-step illustrated cartoon animation on conducting BW. The manual also includes precautions and safety rules to ensure the elderly's safety while performing the exercises. The manual booklet was provided to ensure the participants can refer to and refresh the technical exercise.

### (E) To confirm the time for learning and made sure the trainer guided the participants to learn technical movements of BW.

### Problems anticipated

### The study participants were given a single exercise suit, which came at a reasonably low cost. The funding problem is within the acceptable range of researchers

### Project management

### Training Coach: One coaches and three assistants in experimental groups. One coach and two assistants for each experimental group. One assistant for the control group.

### Coach Qualification: Coaches are physical education teachers, and they have Second-level Social Instructor Certificate and Physical Education Teacher Certificate in the BW group. Coaches are physical education teachers, and they have Second-level Social Instructor Certificate, Physical Education Teacher Certificate. All the assistants are Physical Education Teachers.

### Ethics

### Prior to the commencement of the thesis, ethical permission was obtained from the Medical Research Ethics Committee of Universiti Putra Malaysia (JKEUPM 2020-296).

### Informed consent forms

### Respondent’s Information Sheet and Informed Consent Form (English Version)

###
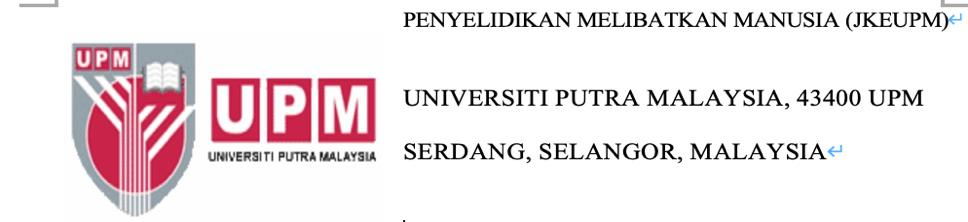


### FORM 2.4: RESPONDENT’S INFORMATION SHEET AND INFORMED CONSENT FORM

### Please read the following information carefully and do not hesitate to discuss any questions you may have with the researcher.

### 1.STUDY TITLE: Effects of brisk walking combined with Tai Chi chuan on health-related physical fitness and selected parameters among older Chinese women

### 2. INTRODUCTION:

### To solve the problems caused by the aging population, the Chinese government has issued three policies since 2016 to pay close attention to the health problems of the elderly and actively organize the elderly to improve their health through exercise. According to the World Health Organization, about 84 % of China's elderly do not exercise regularly. The physical exercise rate of older men is higher than that of women. The prevalence of hypertension in the elderly aged 60 or above in China is 66.9%; The overall prevalence of diabetes was 19.6%. Prevalence of arthritis (30.0%); The prevalence of Alzheimer's disease and other forms of Alzheimer's disease increases with age, and women in these programs have a higher prevalence than men.

### Among them, people aged between 60 and 70 have a 5-13% risk of severe skeletal muscle degeneration (Sakuma, K., & Yamaguchi, A.2012) and the average balance ability decreases by 16% or more every 10 years. The dividing line of human balance ability is 60(King, et al,1994). By 2025, all Chinese cities will be aging, and the population will reach 300 million, and data show that the proportion of the elderly population aged between 60 and 69 years old is the largest (Huang R.,2010). Therefore, women aged 60-69 years old were selected for this study.

### 196 Aerobic exercise is easy to organize such as brisk walking, square dance, Tai Chi, and other group sports are popular among the elderly (Stevens, J. A., & Burns, E. 2015). Tai chi and walking are two projects that middle- aged and old women like and participate in more (Liu, S.J,2013). The function of Tai Chi is diverse, it has fitness, entertainment, acrobatic, medical, artistic and competition are equal to one, it is very worthwhile to practice martial arts antagonism (Fan, Y,2012). Tai Chi, and fitness running have three different fitness methods. The fitness effect has its own focus. Middle-aged and elderly women with different physical conditions should choose according to their own circumstances. Sports, using a variety of sports combined sports methods to make up for the deficiencies between the various sports, play their own characteristics, and ultimately improve fitness (Xuan Lei,2018). So in this study combine brisk walking with Tai chi chuan and exercise with square dancing to explore new ways of fitness for the elderly.

### 3. WHAT WILL YOU HAVE TO DO?

### This study will conduct a test and comparative analysis of the subjects' health related performance variables pre-test and post-test the experiment through 12 weeks of intervention (3times/week) and explore the effects of combination training on health-related physical fitness among Chinese elderly women.

### 4. WHO SHOULD NOT PARTICIPATE IN THE STUDY?

### The exclusion criteria of the samples in this study are: (1) If the subject has recently (less than one year) undergone surgery on the knee, elbow, shoulder, etc., has a history of rheumatoid disease or neurological damage, and is still receiving treatment, it will be excluded; (2) Subjects who were about to stop training due to uncertain factors were directly excluded; (3) Subjects participated in other sports training at the same time.

### 5. WHAT WILL BE THE BENEFITS OF THE STUDY:

### (a) TO YOU AS THE SUBJECT?

### Participating in this experiment will help improve your specific physical fitness and enhance physical fitness and prevent and slow down the occurrence of diseases.

### (b) TO THE INVESTIGATOR?

### Contribute to the development of effective exercise.

### 6. WHAT ARE THE POSSIBLE RISKS?

### There may be adverse reactions during the study. We will monitor any adverse reactions in all subjects during the intervention training. If you have any adverse reactions during the intervention training process, please promptly report to your researcher or coach. At present, related studies have shown that the possible adverse effects of training include muscle soreness, exercise fatigue, etc. These can be alleviated with proper rest and do not require special treatment. If other sports injuries occur, no compensation or treatment will be provided in this study. You need to tell your family or friends close to you that you are participating in the sports intervention study, and they can pay attention to the events described above. If they have questions about your participation in the research, you can tell them how to contact your researcher or coach.

### 7. WILL THE INFORMATION THAT YOU PROVIDE AND YOUR

### IDENTITY REMAIN CONFIDENTIAL?

### Your health record will be kept at University Putra Malaysia. Researchers, research authorities, and ethics committees will be allowed to access your health records. Any public report on the results of this research will not disclose your personal identity. We will make every effort to protect your personal privacy within the scope permitted by law. When you sign this informed consent form, you agree that your personal and health information will be used in the situations described above.

### 8. WHO SHOULD YOU CONTACT IF YOU HAVE ADDITIONAL QUESTIONS DURING THE COURSE OF THE RESEARCH?

### If you have any questions about this research or any physical discomfort during the research, you can always contact the researcher of this research.

### Researcher's name: BAI XIAORONG

### Mob: 601133836641

### E-mail: baixiaorong188@gmail.com

### Please initial here if you have read and understood the contents of this

### page______

### I …………………………………… Identity Card No. …………………………… address………………………………………………………………………………………………………... ……………………………………………………..hereby voluntarily agree to take part in the research stated above *(clinical /drug trial/video recording/ focus group/interview-based/ questionnaire- based).

### I have been informed about the nature of the research in terms of methodology, possible adverse effects and complications (as written in the Respondent’s Information Sheet). I understand that I have the right to withdraw from this research at any time without giving any reason whatsoever. I also understand that this study is confidential, and all information provided with regard to my identity will remain private and confidential.

### I* wish / do not wish to know the results related to my participation in the research

### I agree/do not agree that the images/photos/video recordings/voice recordings related to me be used in any form of publication or presentation

### (if applicable)

### * Delete where necessary

### Signature…….…………………………

### Signature …….………………………

### (Respondent)

### (Witness)

### Date:………………………………….…..

### Name :………………………………….…..

### I/C No. :………………………………….…..

### I confirm that I have explained to the respondent the nature and purpose of

### the above-mentioned research.

### Date …….…………………………

### Signature …….………………………….

**Research protocol: part 2**

### Budget

### The study participants were given a single exercise suit, which came at a reasonably low cost.

### A piece of clothing 50 yuan, a total of 60 pieces, cost 3000 yuan.

### Printing sport booklet costs 2 yuan for each, printing a total of 30 copies, a total of 60 yuan.

### Professional trainers are friends of researchers and do not cost.

### Other support for the project

### This section should provide information about the funding received or anticipated for this project from other funding organizations.

### Collaboration with other scientists or research institutions

### No application.

### Curriculum Vitae of investigators

### Wensheng Xiao, PhD, Lecturer, and Tel: +86 13735107256. He to supervise them to do exercise and guide them to complele training log and check them to rational diet, training site in puyang square, Puyang,Henan province, China.

### Xiaorong Bai, PhD, Lecturer, and Tel: +86 13515728656. She to supervise them to keep daily life routine and check them to rational diet.

### Other research activities of the investigators

### No application.

### Financing and insurance

### In China, every elderly person has their own medical insurance, which can guarantee the smooth conduct of the experiment.

### Reference

### ACSM. (2005). ACSM’s health-related physical fitness assessment manual (G. Davis & S. Dwyer (eds.)). American College of Sports Medicine. http://search.ebscohost.com/login.aspx?direct=true&db=edshlc&AN=edshlc.009344897-X&site=eds-live

### ACSM. (2010). ACSM’s health-related physical fitness assessment manual (L. Kaminsky (ed.)). American College of Sports Medicine. https://doi.org/10.1097/00005768-200409000-00030

### ACSM. (2014). American College of Sports Medicine. Guidelines for Exercise Testing and Prescription , Ninth Edition. Lippincott Willians & Wilkins. https://www.amazon.com/ACSMs-Guidelines-Exercise-Testing-Prescription/dp/1609139550

### Ali, A., Abu Zar, M., Kamal, A., Faquih, A. E., Bhan, C., Iftikhar, W., Malik, M. B., Ahmad, M. Q., Ali, N. S., Sami, S. A., Jitidhar, F., Cheema, A. M., & Zulfiqar, A. (2018). American Heart Association High Blood Pressure Protocol 2017: A Literature Review. Cureus, 10(8), e3230. https://doi.org/10.7759/cureus.3230

### Awick, E. A., Wójcicki, T. R., Olson, E. A., Fanning, J., Chung, H. D., Zuniga, K., Mackenzie, M., Kramer, A. F., & McAuley, E. (2015). Differential exercise effects on quality of life and health-related quality of life in older adults: a randomized controlled trial. Quality of Life Research, 24(2), 455–462. https://doi.org/10.1007/s11136-014-0762-0

### Bai, X., Soh, K. G., Omar Dev, R. D., Talib, O., Xiao, W., & Cai, H. (2022). Effect of Brisk Walking on Health-Related Physical Fitness Balance and Life Satisfaction Among the Elderly: A Systematic Review. In Frontiers in Public Health (Vol. 9, p. 2386). Frontiers Media S.A. https://doi.org/10.3389/fpubh.2021.829367

### Bullo, V., Gobbo, S., Vendramin, B., Duregon, F., Cugusi, L., Di Blasio, A., Bocalini, D. S., Zaccaria, M., Bergamin, M., & Ermolao, A. (2018). Nordic Walking Can Be Incorporated in the Exercise Prescription to Increase Aerobic Capacity, Strength, and Quality of Life for Elderly: A Systematic Review and Meta-Analysis. In Rejuvenation Research (Vol. 21, Issue 2, pp. 141–161). https://doi.org/10.1089/rej.2017.1921

### Choi, M., Prieto-Merino, D., Dale, C., Nüesch, E., Amuzu, A., Bowling, A., Ebrahim, S., & Casas, J. P. (2013). Effect of changes in moderate or vigorous physical activity on changes in health-related quality of life of elderly British women over seven years. Quality of Life Research, 22(8), 2011–2020. https://doi.org/10.1007/s11136-012-0332-2

### Department of Mass Sports, G. A. of S. of C. (2003). National Physical Fitness Standards Manual (Elderly Section). General Administration of Sport of China. http://www.sport.gov.cn/qts/n4986/c670121/content.html

### Fan, B. (2018). Effects of different steps of brisk walking on the fitness of middle-aged and old men. Zhongguo Ying Yong Sheng Li Xue Za Zhi, 34(2), 126–129. https://doi.org/10.12047/j.cjap.5605.2018.000

### Gschwind, Y. J., Kressig, R. W., Lacroix, A., Muehlbauer, T., Pfenninger, B., & Granacher, U. (2013). A best practice fall prevention exercise program to improve balance, strength / power, and psychosocial health in older adults: Study protocol for a randomized controlled trial. BMC Geriatrics, 13(1), 1–13. https://doi.org/10.1186/1471-2318-13-105

### Im, J. Y., Bang, H. S., & Seo, D. Y. (2019). The effects of 12 weeks of a combined exercise program on physical function and hormonal status in elderly Korean women. International Journal of Environmental Research and Public Health, 16(21). https://doi.org/10.3390/ijerph16214196

### Lombardo, P., Jones, W., Wang, L., Shen, X., & Goldner, E. M. (2018). The fundamental association between mental health and life satisfaction: Results from successive waves of a Canadian national survey. BMC Public Health, 18(1), 1–9. https://doi.org/10.1186/S12889-018-5235-X/TABLES/4

### Michalski, C. A., Diemert, L. M., Hurst, M., Goel, V., & Rosella, L. C. (2022). Is life satisfaction associated with future mental health service use? An observational population-based cohort study. BMJ Open, 12, 50057. https://doi.org/10.1136/bmjopen-2021-050057

### Morris, J. N., & Hardman, A. E. (1997). Walking to health. Sports Medicine, 23(5), 306–332. https://doi.org/10.2165/00007256-199723050-00004

### Ordu Gokkaya, N. K., Gokce-Kutsal, Y., Borman, P., Ceceli, E., Dogan, A., Eyigor, S., & Karapolat, H. (2012). Pain and quality of life (QoL) in elderly: The Turkish experience. Archives of Gerontology and Geriatrics, 55(2), 357–362. https://doi.org/10.1016/j.archger.2011.10.019

### Rikli, R., & Jones, C. (2013). Senior fitness test manual. In Human kinetics. (Second Edi). https://doi.org/10.5860/choice.39-3447

### Riva, G., & Serino, S. (2020). Virtual reality in the assessment, understanding and treatment of mental health disorders. Journal of Clinical Medicine, 9(11), 1–9. https://doi.org/10.3390/jcm9113434

### Steven, S. (2021). Ducking and Diving: An evaluation and mapping of mid-career UK actors’ and performers’ career sustaining strategies. In Goldsmiths. [Doctoral dissertation, University of London]. United Kingdom.

### Stewart, R. A. H., Held, C., Hadziosmanovic, N., Armstrong, P. W., Cannon, C. P., Granger, C. B., Hagström, E., Hochman, J. S., Koenig, W., Lonn, E., Nicolau, J. C., Steg, P. G., Vedin, O., Wallentin, L., & White, H. D. (2017). Physical Activity and Mortality in Patients With Stable Coronary Heart Disease. Journal of the American College of Cardiology, 70(14), 1689–1700. https://doi.org/10.1016/j.jacc.2017.08.017

### Swoap, R. A., Norvell, N., Graves, J. E., & Pollock, M. L. (1994). High versus Moderate Intensity Aerobic Exercise in Older Adults: Psychological and Physiological Effects. Journal of Aging and Physical Activity, 2(4), 293–303. https://doi.org/10.1123/japa.2.4.293

### Wang, P., Li, Y., Liu, X., Wang, Q., Guo, Y., Zhao, Y., Li, L., Fan, J., Zhou, H., Mao, Z., Zhang, G., & Wang, C. (2017). Independent and cumulative effects of resting heart rate and pulse pressure with type 2 diabetes mellitus in Chinese rural population. Scientific Reports, 7(1), 1–8. https://doi.org/10.1038/s41598-017-02758-1

### WHO. (2020). Physical activity. World Health Organization. https://www.who.int/news-room/fact-sheets/detail/physical-activity
