# Supplementary material for: Effects of 12-weeks of Brisk Walking on Health-related Physical Fitness, Balance, and Life Satisfaction in Overweight Older Chinese Women: A Cluster Randomized Control Trial
Source: PLoS One. 2026 Jun 26;21(6):e0352243. doi: 10.1371/journal.pone.0352243 (PMC13308794; doi:10.1371/journal.pone.0352243)
Supplement: S3 File — (PDF) [file pone.0352243.s002.pdf]

PEJABAT TIMBALAN NAIB CANSELOR (PENYELIDIKAN DAN INOVASI)

OFFICE OF THE DEPUTY VICE CHANCELLOR (RESEARCH AND INNOVATION

Rujukan kami : UPM/TNCPI/RMC/1.4.18.2 (JKEUPM)

Tarikh : 26 May 2021

Prof. Dr. Soh Kim Geok
Department of Sports Studies
Faculty of Educational Studies
Universiti Putra Malaysia
Serdang, Selangor

Dear Madam/Sir,

RESEARCH PROJECT: EFFECTS OF BRISK WALKING COMBINED WITH TAI CHI CHUAN ON HEALTH-RELATED PHYSICAL FITNESS AND SELECTED HEALTH PARAMETERS AMONG OLDER CHINESE WOMEN.

REFERENCE NO: JKEUPM-2020-296

RESEARCHER : BAI XIAORONG
SUPERVISOR : PROF. DR. SOH KIM GEOK

The Ethics Committee for Research involving Human Subjects of University Putra Malaysia (JKEUPM) has studied the proposal for the above project and found that there were no objectionable ethical issues involved in the proposed study.

Please find the list of documents received and reviewed with reference to the study and committee members who reviewed the documents (as attached).

Notwithstanding above, we will not be responsible for any misconduct on the part of researcher in the course of carrying out the research.

Ethical approval is required in the case of amendments/ changes to the study documents/ study sites/ study team.

Thank you.

"WITH KNOWLEDGE WE SERVE"

Sincerely yours,

PROF. DR. ZAMBERI SEKAWI
Chair
Ethics Committee for Research involving Human Subjects
Universiti Putra Malaysia

ETHICS COMMITTEE FOR RESEARCH INVOLVING HUMAN SUBJECTS (JKEUPM)
UNIVERSITI PUTRA MALAYSIA

**Research title**	**:**	**Effects of Brisk Walking Combined with Tai Chi Chuan on Health-Related Physical Fitness and Selected Health Parameters among Older Chinese Women.**
**Study Site**	**:**	**China**
**JKEUPM Ref No.**	**:**	**JKEUPM-2020-296**
**Researcher**	**:**	**Bai Xiaorong**
**Supervisor**	**:**	**Prof. Dr. Soh Kim Geok**

Documents received and reviewed with reference to the above study:

1. Ethics Application Form, Version 2 dated 22/1/2021
2. Respondent Information Sheet & Consent (English), Version 4 dated 4/5/2021
3. Respondent Information Sheet & Consent (Others), Version 3 dated 4/5/2021
4. Proposal (English), Version 3 dated 4/5/2021
5. Questionnaire/Interview (English), Version 2 dated 22/1/2021
6. Curriculum Vitae of:
   a. Prof. Dr. Soh Kim Geok
   b. Dr. Roxana Dev Omar Dev
   c. Assoc. Prof. Dr. Tengku Fadilah Tengku Kamalden
   d. Bai Xiaorong

The University Research Ethics Committee, Universiti Putra Malaysia (JKEUPM) operates in accordance to the ICH-GCP Guidelines.
Decision by JKEUPM:

[X] Approved

[X] **Permission MUST BE OBTAINED from the respective hospitals/ institutions before conducting the research**

[ ] Disapproved

Please note that the approval is **VALID UNTIL 26 MAY 2022**

Researchers should comply with the following:

I. Complete a Study Final Report upon study completion (Form 3.2).

II. Ethical approval is required in the case of amendments/ changes to the study documents/ study sites/ study team.

III. Applicable for Clinical Trial Studies and Clinical interventional Studies only: Progress Report has to be submitted to JKEUPM at every 6 months from the date of approval (Form 3.1). Report occurrences of all Serious Adverse Events (SAEs), Suspected Unexpected Serious Adverse Reaction (SUSARs) and Protocol Deviation/ Violation at all JKEUPM approved sites to JKEUPM. SAEs are to be reported within 15 calendar days from awareness of event by

investigator. Initial report of SUSARs are to be reported as soon as possible but not later than 7 calendar days from awareness of event by investigator, followed by a complete report within 8 additional calendar days.

The required forms can be obtained from the Ethics Committee for Research Involving Human Subjects (JKEUPM) website (http://www.tncpi.upm.edu.my/faildokumen).

Date of Approval: 26 May 2021

Members of the JKEUPM who reviewed the documents:

i. Primary Reviewer: Assoc. Prof. Dr. Nur Surayyah Madhubala Abdullah
ii. Informed Consent Reviewer: Dr. Nur Indah Ahmad

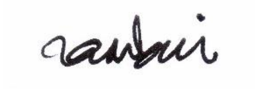

……………………………………………

PROF. DR. ZAMBERI SEKAWI
Chair
Ethics Committee for Research involving Human Subjects
Universiti Putra Malaysia

JKEUPM is recognised by The Strategic Initiative for Developing Capacity in Ethical Review (SIDCER) in collaboration with the Forum for Ethical Review Committees in Asia and the Western Pacific Region (FERCAP) for its compliance with the Declaration of Helsinki, International Conference on Harmonization (ICH) Guidelines, Good Clinical Practice (GCP) Standards, Council for International Organizations of Medical Sciences (CIOMS) Guidelines, World Health Organization (WHO) Standards and Operational Guidance for Ethics Review of Health-Related Research and Surveying and Evaluating Ethical Review Practices, EC/IRB Standard Operating Procedures (SOPs), and Local Regulations and Standards in Ethical Review.

Approved at JKEUPM Meeting on 25 August 2020, attended by:

NAME	DESIGNATION	GENDER	TICK IF PRESENT
Prof. Dr. Zamberi Sekawi	Professor of Medical Microbiology, Department of Medical Microbiology and Parasitology, Faculty of Medicine and Health Sciences.	Male	√
Prof. Dr. Johnson Stanslas	Professor of Pharmacology, Department of Medicine, Faculty of Medicine and Health Sciences.	Male	√
Prof. Dr. Rukman Awang Hamat	Professor of Clinical Microbiology, Department of Medical Microbiology and Parasitology, Faculty of Medicine and Health Sciences.	Male	√
Prof. Dr. Normala Ibrahim	Associate Professor of Psychiatry, Department of Psychiatry, Faculty of Medicine and Health Sciences.	Female	
Assoc. Prof. Dr. Wan Aliaa Wan Sulaiman	Associate Professor of Neurology, Department of Medicine, Faculty of Medicine and Health Sciences.	Female	√
Assoc. Prof Dr. Shamala Paramasivam	Associate Professor of English Language, Department of English, Faculty of Modern Languages and Communication.	Female	
Assoc. Prof. Dr. Hayati Kadir@Shahar	Senior Lecturer of Public Health Medicine Specialist, Department of Community Health, Faculty of Medicine and Health Sciences	Female	√
Assoc. Prof. Dr. Salmiah Md. Said	Senior Lecturer of Public Health Medicine Specialist, Department of Community Health, Faculty of Medicine and Health Sciences	Female	√
Assoc Prof. Dr. Rosliza Abdul Manaf	Associate Professor of Public Health Medicine Specialist, Department of Community Health, Faculty of Medicine and Health Sciences.	Female	√
Assoc. Prof. Dr. Nur Surayyah Madhubala Abdullah	Senior Lecturer of Moral and Citizenship Education, Department of Language and Humanities Education, Faculty of Educational Studies	Female	√
Assoc. Prof. Dr. Syamsiah Mashohor	Associate Professor of Computer Engineering, Department of Computer and Communication Systems, Faculty of Engineering	Female	
Dr. Zatul Himmah Binti Adnan	Lecturer of Politics and Policy, Department of Government and Civilisation Studies, Faculty of Human Ecology	Female	√
Assoc. Prof. Dr Chew Boon How	Senior Lecture of Family Medicine Specialist, Department of Family Medicine, Faculty of Medicine and Health Sciences.	Male	√
En. Ahmad Jamil Mahabob	Retired Government Staff	Male	√
Pn. Mimi Nora Binti Mansor (Independent Member/Layperson)	Retired Government Staff	Female	√
Assoc. Prof. Dr. Thilakavathy Karuppiah			
